# The *Pseudomonas putida* T6SS is a plant warden against phytopathogens

**DOI:** 10.1038/ismej.2016.169

**Published:** 2017-01-03

**Authors:** Patricia Bernal, Luke P Allsopp, Alain Filloux, María A Llamas

**Affiliations:** 1 MRC Centre for Molecular Bacteriology and Infection, Department of Life Sciences, Imperial College London, London, UK; 2 Department of Environmental Protection, Estación Experimental del Zaidín-Consejo Superior de Investigaciones Científicas, Granada, Spain

## Abstract

Bacterial type VI secretion systems (T6SSs) are molecular weapons designed to deliver toxic effectors into prey cells. These nanomachines have an important role in inter-bacterial competition and provide advantages to T6SS active strains in polymicrobial environments. Here we analyze the genome of the biocontrol agent *Pseudomonas putida* KT2440 and identify three T6SS gene clusters (K1-, K2- and K3-T6SS). Besides, 10 T6SS effector–immunity pairs were found, including putative nucleases and pore-forming colicins. We show that the K1-T6SS is a potent antibacterial device, which secretes a toxic Rhs-type effector Tke2. Remarkably, *P. putida* eradicates a broad range of bacteria in a K1-T6SS-dependent manner, including resilient phytopathogens, which demonstrates that the T6SS is instrumental to empower *P. putida* to fight against competitors. Furthermore, we observed a drastically reduced necrosis on the leaves of *Nicotiana benthamiana* during co-infection with *P. putida* and *Xanthomonas campestris*. Such protection is dependent on the activity of the *P. putida* T6SS. Many routes have been explored to develop biocontrol agents capable of manipulating the microbial composition of the rhizosphere and phyllosphere. Here we unveil a novel mechanism for plant biocontrol, which needs to be considered for the selection of plant wardens whose mission is to prevent phytopathogen infections.

## Introduction

**Figure 1:**
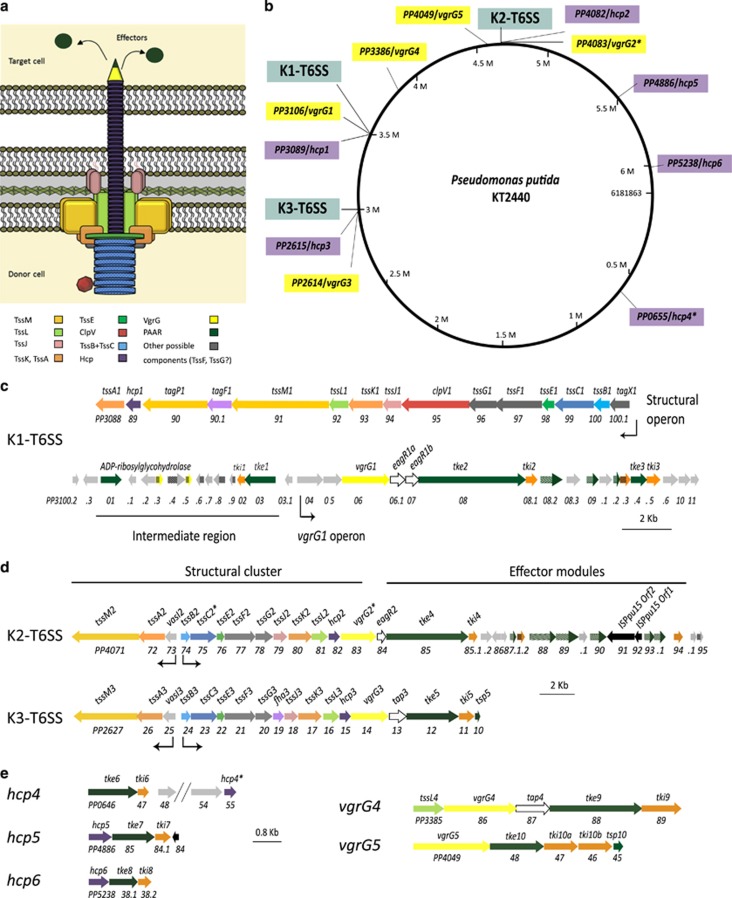
T6SS clusters in *P. putida* KT2440. (**a**) Schematic representation of the T6SS structure. (**b**) Distribution of the K1-, K2- and K3-T6SS clusters (blue), and the *vgrG* (yellow) and *hcp* (purple) genes in the KT2440 genome. (**c**–**e**) Genomic organization of the *P. putida* T6SSs cluster, including K1 (**c**), K2 and K3 (**d**) or the *vgrG* and *hcp* orphan clusters (**e**). The color code of the genes correlates with the color code shown in **a**. The asterisk (*) in the *tssC2*, *vgrG2* and *hcp4* genes indicates that these genes contain premature stop codons.

The type VI secretion system (T6SS) is found in more than 25% Gram-negative bacteria and used to inject toxic proteins into prokaryotic or eukaryotic cells (Ho *et al.*, 2013). Initially, the T6SS was assigned a role in virulence and eukaryotic cell manipulation (Ma and Mekalanos, 2010; Miyata *et al.*, 2011). Further analyses showed that this system has a key role in inter-bacterial competition (Ho *et al.*, 2013). It provides selective advantages to producer strains by annihilating competitors either in an indiscriminate manner or in response to danger signals (Hood *et al.*, 2010; Basler *et al.*, 2013; Ho *et al.*, 2013; Hachani *et al.*, 2014). The T6SS toxins are usually produced together with immunity proteins that prevent self-intoxication. In molecular terms, the T6SS displays structural similarities with the tail and the puncturing device of the bacteriophage T4 (Leiman *et al.*, 2009; Filloux, 2011; Cascales and Cambillau, 2012). It is composed by 13 core components of which some have now been assigned clear functions (Figure 1a). TssB and TssC form a contractile sheath that encases a tube formed by rings of Hcp hexamers (Kudryashev *et al.*, 2015). A puncturing device made up of a trimeric VgrG spike is placed on top of the Hcp tube and crowned with a PAAR protein (Cascales and Cambillau, 2012; Shneider *et al.*, 2013). The cytosolic part of the T6SS docks onto a membrane complex (TssLMJ) probably by interacting with a phage baseplate-like structure (Durand *et al.*, 2015; Filloux and Freemont, 2016; Planamente *et al.*, 2016). On contraction of the TssBC sheath, the T6SS effectors are propelled out of the bacterium together with the Hcp and VgrG proteins and delivered into prey cells (Basler and Mekalanos, 2012). Finally, the ClpV ATPase binds the contracted sheath for disassembly and recycling, enabling another round of injection (Kapitein *et al.*, 2013; Kube *et al.*, 2014). The T6SS is usually quite modular and can accommodate different combinations of VgrG/PAAR proteins to form the tip. This modularity allows the delivery of a great variety of effectors (Shneider *et al.*, 2013; Hachani *et al.*, 2014; Whitney *et al.*, 2014). Alternatively, the effectors can also be ushered in and delivered by the tube-forming Hcp protein (Silverman *et al.*, 2013). Thus, a wide variety of effectors with a broad range of activities can be secreted by a single T6SS. T6SS effectors have been classified into specialized and cargo effectors (Cianfanelli *et al.*, 2016). Specialized effectors are domains, usually at the C-terminus of specific T6SS structural components that are coined as ‘evolved' VgrG, PAAR or Hcp proteins. In contrast, cargo effectors interact non-covalently with ‘canonical' VgrG, PAAR or Hcp proteins (Durand *et al.*, 2014). Several cargo effectors carry a motif named MIX (marker for type six effectors) that is proposed to be required for recognition and direct interaction with VgrG or PAAR proteins (Salomon *et al.*, 2014). Specific adaptors such as Tap/Tec and EagR proteins are likely to facilitate the interaction between the structural components of the T6SS tip and the effectors (Alcoforado Diniz and Coulthurst, 2015; Liang *et al.*, 2015; Unterweger *et al.*, 2015). Finally, accessory elements (named *tag* from T6SS accessory genes) are required to modulate the assembly of the system and/or confer additional functions (Boyer *et al.*, 2009).

The T6SS was first identified in two pathogenic bacteria, *Vibrio cholerae* and *Pseudomonas aeruginosa* (Mougous *et al.*, 2006; Pukatzki *et al.*, 2006) and analyzed later in many other pathogens (Suarez *et al.*, 2008; de Pace *et al.*, 2010; Burtnick *et al.*, 2011; Murdoch *et al.*, 2011; Rosales-Reyes *et al.*, 2012; Lin *et al.*, 2013). However, analytical description of T6SS in non-pathogenic bacteria is underrepresented in the literature (Bladergroen *et al.*, 2003; Marchi *et al.*, 2013), despite an even distribution in both classes of organisms (Boyer *et al.*, 2009). *Pseudomonas putida* is a saprophytic soil bacterium that has the capacity to colonize the root of crop plants (Espinosa-Urgel *et al.*, 2000; Molina *et al.*, 2000). It is a well-established biocontrol agent that provides growth advantages to the plant (Weller, 2007). In this study we identified and characterized the *P. putida* T6SS, which exhibits great variety and complexity both in terms of apparatus and secreted toxins. We showed that this secretion system is used by the bacterium to drive killing of resilient phytopathogens and appears to be a major factor in its biocontrol portfolio.

## Materials and methods

### Bacterial strains and growth conditions

Bacterial strains are listed in Supplementary Table S1. *P. putida* mutants were constructed by allelic exchange as described previously (Vasseur *et al.*, 2005). Briefly, 750 bp DNA fragments upstream and downstream the gene to be deleted were amplified using KT2440 genomic DNA. Mutator fragments were obtained by overlapping PCR, cloned into pCR-BluntII-TOPO (Invitrogen, ThermoFisher Scientific, Loughborough, UK), sequenced and subcloned into the pKNG101 suicide vector (Kaniga *et al.*, 1991). A similar approach was used to replace the wild-type *tke2* gene with *tke2*-V5 encoding a C-terminally virus 5 (V5)-tagged Tke2 protein. The *hcp1*-HA gene encoding a C-terminal hemagglutinin (HA)-tagged Hcp1 protein was introduced on the chromosome using the miniCTX transposon (Hoang *et al.*, 2000). Insertions and gene replacements were confirmed by PCR. All strains were grown in lysogeny broth (LB; Sambrook *et al.*, 1989). For secretion assays, tryptone soya broth medium (Oxoid, ThermoFisher Scientific, Loughborough, UK) was used. *Escherichia coli* was incubated at 37 °C, and *P. putida* and the phytopathogens at 25–30 °C. Antibiotics were used at (μg ml^−1^): ampicillin, 100; gentamycin, 20; kanamycin, 50; piperacillin, 25; rifampicin, 20; streptomycin, 100; tetracycline, 50.

### Plasmids and cloning

Plasmids are described in Supplementary Table S1 and primers listed in Supplementary Table S2. PCR amplifications were performed using Phusion Hot Start High-Fidelity (Finnzymes, ThermoFisher Scientific, Loughborough, UK), KOD Hot Start (EMD Millipore, Watford, UK) or Taq (Roche, Burgess Hill, UK) DNA polymerases. Recombinant plasmids were sequenced and transferred to *P. putida* by electroporation (Choi *et al.*, 2006) or conjugation (Ramos-Gonzalez *et al.*, 1991).

### Bioinformatic analyses

*Pseudomonas* sequences were obtained from the Pseudomonas Genome database (Winsor *et al.*, 2016). BLASTP analyses were performed at the NCBI website (Boratyn *et al.*, 2013) and amino acid sequence searches using SMART (Letunic *et al.*, 2015) and Pfam (Finn *et al.*, 2016). The Protein Homology/analogy Recognition Engine (Phyre2) server was used to perform structural-base homology prediction (Kelley *et al.*, 2015). The PyMOL Molecular Graphics System (Version 1.8 Schrondinger, LLC, Cambridge, UK) was used to build structural alignments. The phylogenetic tree was constructed using MEGA6 (Tamura *et al.*, 2013). PSORTb software and SOSUI GramN server were used to predict sub-cellular location of proteins (Imai *et al.*, 2008; Yu *et al.*, 2010), TMHMM software to predict transmembrane domains (Krogh *et al.*, 2001), and SignalP and SOSUIsignal to predict signal peptides (Gomi *et al.*, 2004; Petersen *et al.*, 2011). Synteny was analyzed using the CoGe's Genome Evolution tool (Lyons and Freeling, 2008). The UGENE bioinformatics software was used to identify open reading frames (*orfs*; Okonechnikov *et al.*, 2012).

### Secretion assays

Bacterial strains were grown in tryptone soya broth for 5 h at 30 °C and the extracellular fraction obtained and analyzed as previously described (Hachani *et al.*, 2011). The proteins in the culture supernatants were precipitated with trichloroacetic acid and resuspended in 1 M of Tris-base and 4 × Laemmli buffer. Proteins were separated by SDS–polyacrylamide gel electrophoresis containing 8% or 15% (w/v) acrylamide and electro-transferred to nitrocellulose membranes. Immunodetection was performed using monoclonal antibodies directed against the influenza HA epitope (HA.11, Covance, Biolegend, London, UK) or the paramyxovirus of simian V5 epitope (Invitrogen, ThermoFisher Scientific). A monoclonal antibody against the β-subunit of the RNA polymerase (Neoclone, Biolegend, London, UK) was also used. The secondary antibody, horseradish peroxidase-conjugated rabbit anti-mouse (Sigma Aldrich, Gillingham, UK), was detected using the SuperSignal West Pico Chemiluminescent Substrate (Thermo Scientific, Loughborough, UK). Blots were scanned and analyzed using the Image Reader LAS-3000 (Fuji, GE Healthcare, Little Chalfont, UK).

### Growth inhibition assays

Overnight LB cultures of *E. coli* DH5α harboring the vectors pNDM220 (Gotfredsen and Gerdes, 1998), pBAD33 (Guzman *et al.*, 1995) or derivatives encoding Tke2 or Tki2 were adjusted to OD_600_ of 0.1. Expression of *tke2* and *tki2* was induced with 0.2% (w/v) L-arabinose and 1 mM isopropyl β-D-1-thiogalactopyranoside, respectively.

### Interbacterial competition assays

*In vitro* competition assays were performed on LB plates as previously described (Hachani *et al.*, 2013). Bacterial overnight cultures were adjusted to OD_600_ of 1 in PBS and mixed in a 1:1 ratio (*P. putida*-prey). Bacteria were co-cultured at 30 °C for 5 h (*E. coli*) or 24 h (phytopathogens). The competition was quantified by counting colony-forming units on antibiotic selection. At least three biologically independent experiments were performed. *In planta* competition assays were carried out by infiltration of bacteria into *Nicotiana benthamiana* leaves as described before (Ma *et al.*, 2014). Overnight cultures of *P. putida* and *Xanthomonas campestris* were adjusted to OD_600_ of 0.1 in PBS and mixed in a 1:1 ratio. Approximately 100 μl volume was infiltrated on the reverse of a 1-month-old leaf and the infiltration area marked. After 24 h of incubation in a plant chamber (23 °C, 16 h light), colony-forming units were determined. A section of the leaf from the infiltration area was cut out, homogenized in PBS and subsequently serially diluted. The leaves were visualized by fluorescence microscopy using a Leica M206FA stereomicroscope. Imaging was performed at room temperature with a × 1 objective. The evaluation of necrosis was based on the coloration of the leaves following previous standard evaluation of virulence that goes from no visible effects to changes in the tissue color of the leaf, which can shift from green to yellowish (chlorosis), yellowish to brownish and blackening of the leaf (necrosis), up to complete rotting of the leaf at later stages (Katzen *et al.*, 1998). In our assay, the circled zones point at deep brown color area.

## Results

### Genome-wide screening for T6SSs in *P. putida* species

**Figure 2:**
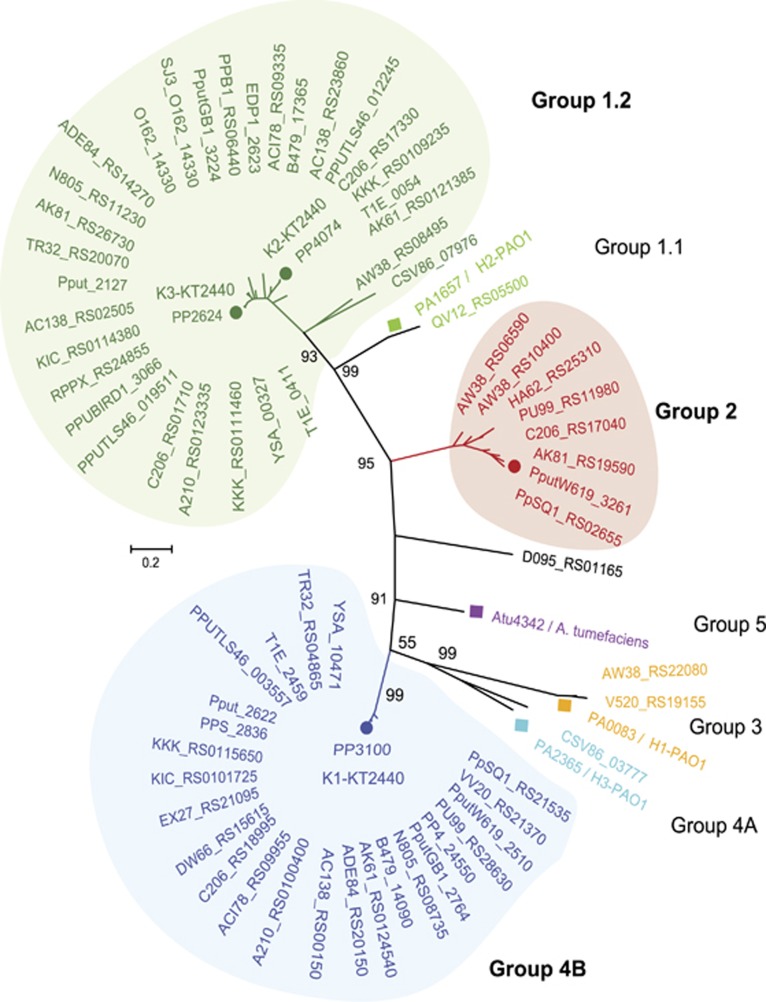
Phylogenetic distribution of T6SS clusters in *P. putida* species. Maximum likelihood tree with 1000 bootstrap replicates were built with Mega 6 for the core component protein TssB. T6SS cluster nomenclature (Boyer *et al.*, 2009; Barret *et al.*, 2011) is used to show the major phylogenetic clusters. Three main groups are clearly distinguishable: group 1.2 (green), group 2 (red) and group 4B (blue). *P. aeruginosa* and *A. tumefaciens* T6SSs loci are included into the phylogenetic tree to illustrate all the subgroups: 1.1 (*P. aeruginosa* H2), 1.2 (*P. putida* K2-K3), 2 (*P. putida* W619), 3 (*P. aeruginosa* H1), 4A (*P. aeruginosa* H3), 4B (*P. putida* K1) and 5 (*A. tumefacines*).

*In silico* analyses of forty *P. putida* strains revealed that all encode T6SS genes and 90% of them have at least one cluster encoding a full set of T6SS components (Supplementary Table S3). The number of T6SS clusters in a single strain ranged from zero in *P. putida* BIRD-1 or H8234 to four in *P. putida* PA14H7, whereas most strains contained one or two clusters (Supplementary Table S3). In total, we identified 66 complete T6SS gene clusters distributed in three main phylogenetic clades (Figure 2). Following previous nomenclature (Boyer *et al.*, 2009; Barret *et al.*, 2011), we referred to these three groups as 1.2, 2 and 4B. Eighty percent of the clusters belong to group 1.2 or 4B, whereas 10% are found in group 2 (Figure 2). Each of these groups contains distinguishable genetic architecture and features (Supplementary Figure S1), as described in the next section.

### The reference strain *P. putida* KT2440 is equipped with three T6SSs

We used the strain KT2440 to perform in-depth genomic analysis. In this strain, only five T6SS-related genes, that is, the *hcp* genes *PP2615* and *PP4082* or the *vgrG* genes *PP2614*, *PP3386* and *PP4049* are annotated (http://pseudomonas.com/). Using bioinformatics approaches (for example, BLASTP, Ugene or SMART) we identified a large number of T6SS-related *orf*s (Supplementary Tables S4–S6). Most of the genes fall into three clusters that we named K1-, K2- and K3-T6SS (Figures 1b–d and Supplementary Tables S4 and S5). Several *hcp* and *vgrG* orphan genes were also found scattered on the chromosome (Figures 1b and e, and Supplementary Table S6). Phylogenetic analysis showed that the K2- and K3-T6SSs are related (group 1.2, Figure 2), whereas the K1 cluster clades separately (group 4B, Figure 2).

K2 and K3 consist of two divergently transcribed gene clusters that contain 12 of the 13 genes encoding core T6SS components (Figure 1d). The missing core gene, *clpV*, encodes the ATPase required for disassembling the sheath (Kapitein *et al.*, 2013; Kube *et al.*, 2014), which is absent in all clusters belonging to group 1.2 (data not shown). Using the ‘CoGe's Genome Evolution Analysis' tool, we observed a synteny among the K2 and K3 clusters (Figure 1d). The identity of the corresponding proteins encoded within each of these clusters was remarkably high, ranging from 64 to 99% (Supplementary Table S5). These observations indicate that the two clusters may have arisen from a duplication event.

The K1 system is not related to K2 and K3, and belongs to the plant-related group (group 4B, Figure 2; Boyer *et al.*, 2009). This cluster comprises two putative operons and an ‘intermediate' region (Figure 1c). The first operon contains 15 genes, 12 of which encode T6SS core components, and was named the structural operon (Figure 1c). The last core component gene, *vgrG*, is located within the second operon that was therefore named the VgrG1 operon (Figure 1c). Within the structural operon we found a previously undefined *orf*, *PP3090.1* encoding the accessory protein TagF1 (Supplementary Table S4). An ortholog of this protein was reported to function as a posttranscriptional regulator (Silverman *et al.*, 2011). Another accessory gene encodes TagP1 (Supplementary Table S4), a TssM derivative whose C-terminal periplasmic portion carries a peptydoglycan-binding domain (pfam00691; Aschtgen *et al.*, 2010). Finally, our analysis identified a novel T6SS feature represented by the first gene in the K1-T6SS structural operon, *PP3101.1*, *tagX1* (Figure 1c and Supplementary Table S4). The protein encoded by this gene has no homologs or recognizable features. It has not been assigned a role in the T6SS but is exclusively present in all clusters belonging to the 4B group (that is, *P. putida* and *Pseudomonas syringae*).

### The K1-T6SS is functional and anti-bacterial

**Figure 3:**
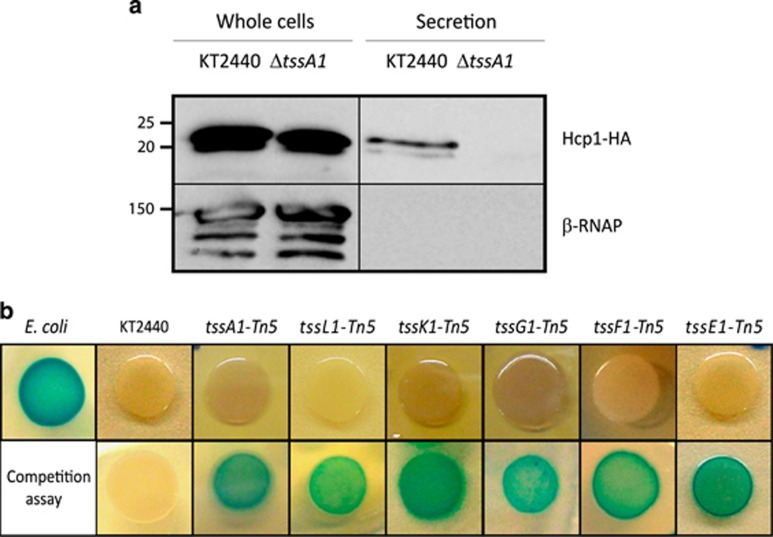
Functionality of the *P. putida* K1-T6SS. (**a**) Production and secretion of Hcp1 in the *P. putida* KT2440 wild type and the Δ*tssA1* mutant strains. The HA-tagged Hcp1 protein was detected by western blot analysis using an anti-HA antibody. Detection of the β-subunit of the RNA polymerase (β-RNAP) was used as control. The position of the molecular size marker (in kDa) is indicated. (**b**) Competition assay between *P. putida* and a *lacZ*-encoding *E. coli* strain. Blue patches on X-gal-containing LB plates indicate *E. coli* survival. The top row shows the growth of *E. coli*, *P. putida* KT2440 wild-type strain and a battery of *P. putida* mutants in K1-T6SS genes. The bottom row shows the growth of mixed *E. coli*/*P. putida* cultures after 5 h of co-incubation.

Hcp release is dependent on the T6SS and is a reliable marker for assessing functionality of the system (Pukatzki *et al.*, 2006). Therefore, we engineered *P. putida* strains producing an HA-tagged version of Hcp1 to assess K1-T6SS activity. TssA is a core baseplate component of the T6SS, is essential for T6SS activity (Planamente *et al.*, 2016) and we used a *tssA* mutant to disable the *P. putida* K1-T6SS. We readily detected Hcp1 in the supernatant of wild-type cultures but not in an isogenic *tssA1* mutant (Figure 3a), thus establishing that the K1-T6SS is a functional secretion machine.

Several characterized T6SSs have anti-bacterial activity, resulting from the injection of T6SS toxins into bacterial preys (Russell *et al.*, 2014; Cianfanelli *et al.*, 2016). We performed competition assays using *E. coli* K12 as prey and *P. putida* wild type or T6SS mutants as predators. The *E. coli* prey harbors a plasmid that confers blue color to the colony in the presence of X-gal (Figure 3b). In a mixed culture, the *P. putida* wild-type strain was able to annihilate *E. coli*, whereas mutants in any of the K1-T6SS structural genes (*tssA1*, *tssL1*, *tssK1*, *tssG1*, *tssF1* or *tssE1*) were no longer outcompeting *E. coli* (Figure 3b). In contrast, mutants in the K2- or K3-T6SS clusters, *P. putida* Δ*tssM2* and Δ*tssM3*, respectively, still efficiently annihilated *E. coli* (data not shown). We concluded that K1 is the most active KT2440 T6SS *in vitro*, as under the laboratory conditions used here, and that its antibacterial activity may result from the secretion of T6SS effectors.

### *P. putida* KT2440 encodes a wealth of T6SS bacterial effectors

**Figure 4:**
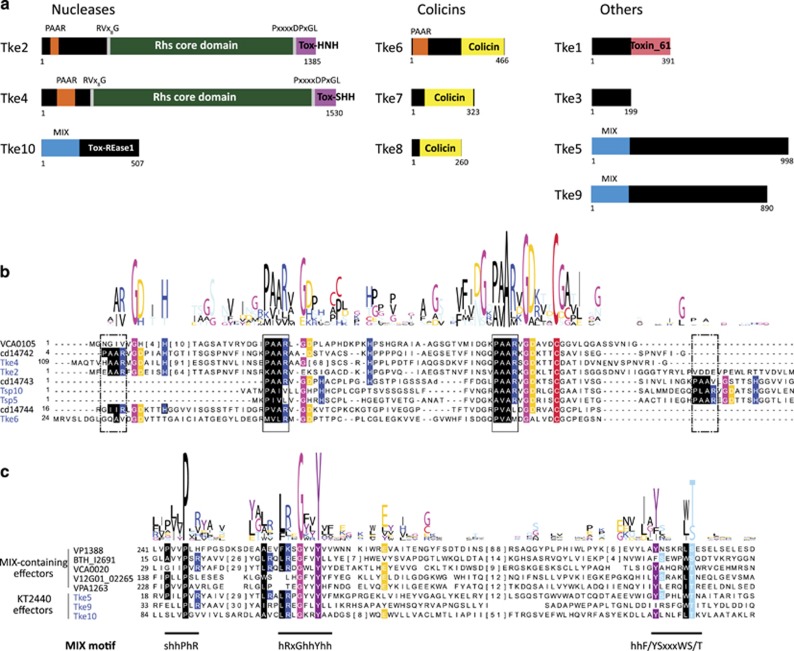
*P. putida* KT2440 T6SS effectors. (**a**) The domain organization of the putative effectors is shown, with PAAR motifs indicated in orange, MIX motifs in blue, Rhs domains in green, HNH nuclease motifs (Tox-HNH and Tox-SHH) in purple, colicin motifs in yellow and the Tox-61 domain in pink. Multiple sequence alignments of the PAAR (**b**) and MIX (**c**) protein motifs are represented. The KT2440 T6SS effectors identified in this work are indicated in blue. The sequence of known T6SS effectors containing these motifs was retrieved from the NCBI database (http://www.ncbi.nlm.nih.gov/Structure/cdd/cdd.shtml). Conservation logos of the motifs are indicated above the alignment. Conserved residues are highlighted according to the amino acid characteristic: hydrophobic (black), small (pink), positive (blue), negative (yellow) and polar (purple, light blue, red).

Genes encoding putative T6SS effectors and cognate immunity proteins (effector–immunity (EI) pairs) are often linked to *hcp*, *vgrG* genes and/or genes encoding chaperones/adaptors (Dong *et al.*, 2013; Hachani *et al.*, 2014; Ma *et al.*, 2014; Liang *et al.*, 2015; Unterweger *et al.*, 2015). Our *in-silico* analyses identified a total of 10 potential EI pairs, most of them encoded in the vicinity of *vgrG*/*hcp* genes and in some cases near genes encoding Tap or EagR adaptors (Figures 1c–e and Supplementary Tables S4–S6). These EI pairs have been named Tke and Tki for Type six KT2440 effector and immunity, respectively (Figure 4a).

#### VgrG linked effectors

**Figure 5:**
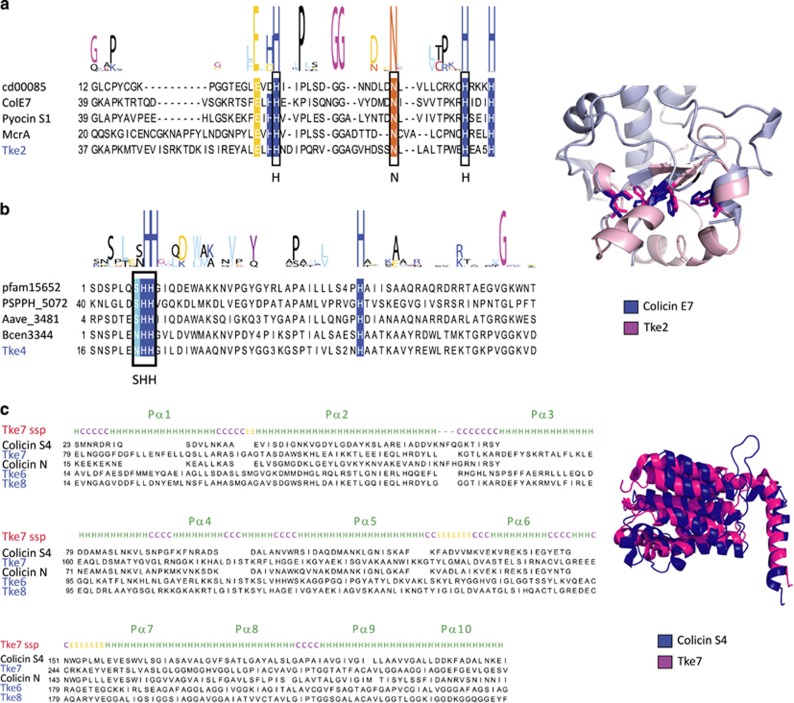
*P. putida* KT2440 T6SS nucleases. (**a**, **b**) Multiple sequence alignments of the C-terminal domains of Tke2 (**a**) and Tke4 (**b**) effectors (blue) with known proteins of the family (black). Conservation logos of the motifs HNH (**a**) and SHH (**b**) are indicated above the alignment. Conserved residues are indicated with the color code used in Figure 4. A representation of the structural model of the C-terminal domain of the Tke2 effector (magenta) superimposed on the colicin E7 structure (blue; PDB: 2JB0) is shown on the right of **a**. Side chains of the active site residues are shown. (**c**) Multiple sequence alignment of T6SS colicin effectors (blue) with known proteins of the family (black). The secondary structure prediction (ssp) for effector Tke7 is shown above the alignment. A structural alignment of the Tke7 effector model (magenta) with the colicin S4 (blue, PDB: 3FEW) is shown on the right.

Downstream *vgrG1* and *vgrG2* in the K1 and K2 clusters, respectively, putative effector genes, *tke2* and *tke4*, and EagR adaptor genes, *eagR1a*-*eagR1b* and *eagR2*, were found (Figures 1c and d). Tke2 and Tke4 proteins share a similar structure, both containing an N-terminal PAAR motif (Cascales and Cambillau, 2012; Shneider *et al.*, 2013) and a conserved Rhs domain (Busby *et al.*, 2013) limited by specific RVxxxxxxxxG and PxxxxDPxGL motifs (Figures 4a and b). PAAR proteins have been shown to be located at the tip of the VgrG trimer, sharpening the T6SS spike and/or creating an interface for T6SS effectors and adaptors (Whitney *et al.*, 2015). The C-terminal region of Tke2 or Tke4 (110 and 102 amino acid long, respectively) carries a cytotoxic domain. This domain is similar in both proteins and belongs to the HNH superfamily of nucleases, for example, colicin E7 and pyocin S1 (Figures 4a and 5a; Huang and Yuan, 2007), although Tke4 domain contains a specific SHH signature (Figures 4a and 5b). Genes encoding putative effectors were also found downstream *vgrG3*, *vgrG4* and *vgrG5* (Figures 1d and e). The *tke5* and *tke9* genes within the K3 and *vgrG4* operons, respectively, are linked to genes encoding Tap adaptors (*tap3* and *tap4*; Figures 1d and e). No recognizable features were found in Tke5 or Tke9, except for a conserved N-terminal MIX motif considered a marker for T6SS effectors (Salomon *et al.*, 2014; Figures 4a and c). This motif is also present in the effector-encoded downstream *vgrG5*, Tke10, which is predicted to be a restriction endonuclease (Figures 1e and 4c). In addition, *tke5* and *tke10* are linked to genes encoding a PAAR-motif (named *tsp* for *type six paar*; *tsp5* and *tsp10*; Figures 1d, e and 4b, and Supplementary Table S6).

#### Effectors encoded in proximity to *hcp* genes

The potential effector genes *tke6*, *tke7* and *tke8* were found within or in the vicinity of the three *hcp* orphan operons (*hcp4*, *hcp5* and *hcp6*; Figure 1e and Supplementary Table S6). These effectors have similarities with pore-forming colicins (that is, colicin S4; Figures 4a and 5c, and Supplementary Table S6). The *tke7* and *tke8* genes are not genetically associated with *vgrG* or T6SS adaptor genes. These *hcp*-linked T6SS effectors could be delivered by docking into the lumen of the Hcp ring, instead of being attached at the VgrG tip, as observed with the *P. aeruginosa* Tse2 effector (Silverman *et al.*, 2013). In contrast to *tke7* and *tke8*, *tke6* is not located within the *hcp* operon but 5 kb upstream of the *hcp4* gene. Interestingly, *hcp4* has a premature stop codon and might not be functional (Figure 1e and Supplementary Table S6), whereas Tke6 has an N-terminal PAAR domain (Figures 4a and b). Thus, in contrast to Tke7 and Tke8 that lack PAAR or MIX domains, the delivery of Tke6 could be mediated by a VgrG protein through a PAAR-VgrG interaction.

#### Orphan effectors

We found two additional potential EI pairs (*tke1-tki1* and *tke3-tki3*) within the K1-T6SS cluster, both lacking PAAR or MIX motifs. Tke1 is an ortholog of the *P. aeruginosa* Tse6, which presents a C-terminal region carrying a toxic domain known as Toxin_61 (Figure 4a and Supplementary Figure S2a; Hachani *et al.*, 2014; Whitney *et al.*, 2014) and degrades NAD(P)(+) in target cells (Whitney *et al.*, 2015). In case of Tke3, a Phyre2 analysis suggests that the C-terminal domain resembles the B30.2 fragment from the human protein TRIM20 (Weinert *et al.*, 2015; Supplementary Figure S2b and Supplementary Table S4).

In summary, we identified 10 potential T6SS effectors in the KT2440 genome. Three of them Tke2, Tke4 and Tke6 have an N-terminal PAAR domain (Figures 4a and b) and are therefore considered ‘specialized' effectors. The others are not fused to any T6SS component and their domain architecture suggests they are ‘cargo' effectors.

### Tke2/Tki2 is a *P. putida* K1-T6SS effector/immunity pair

**Figure 6:**
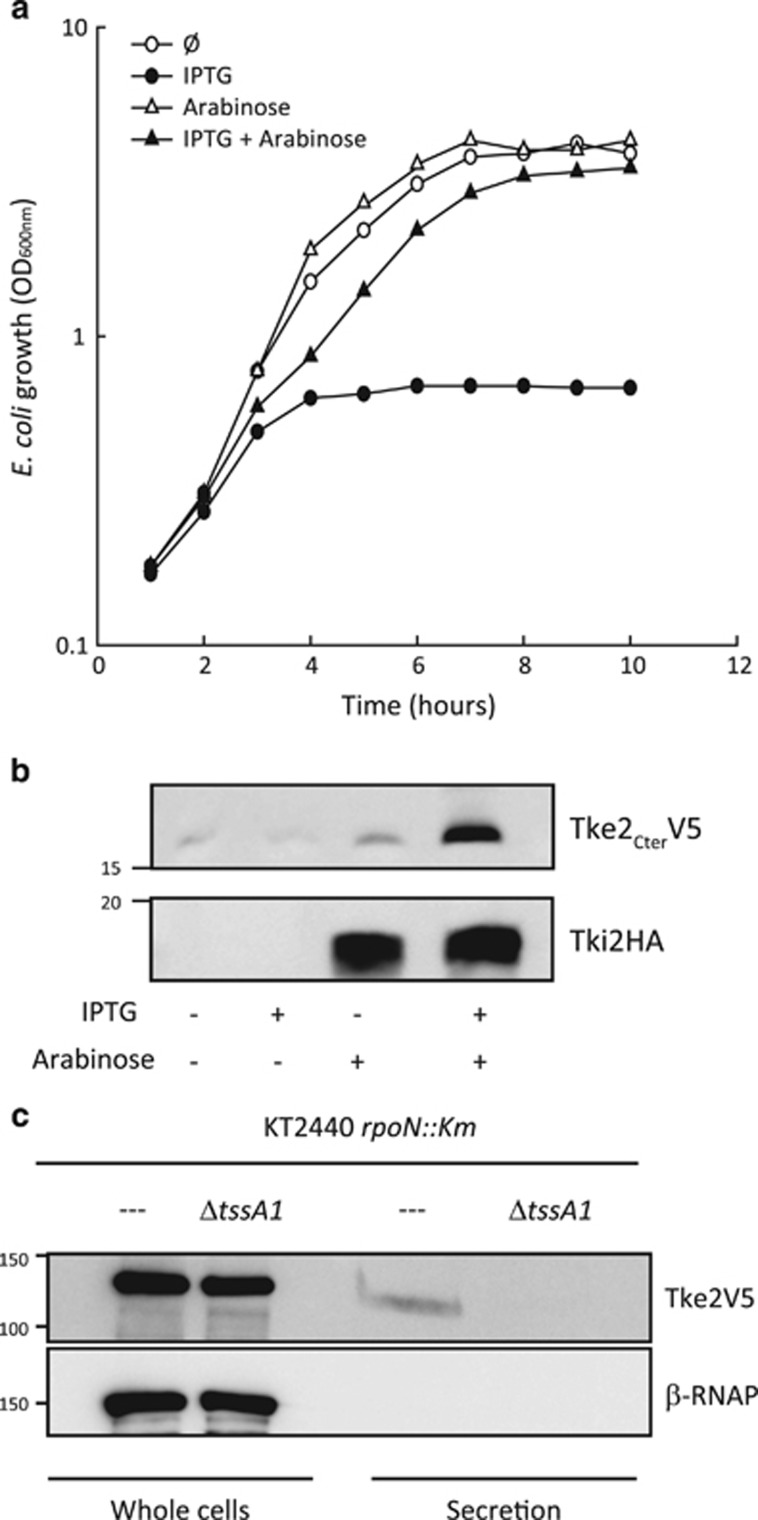
Toxicity and secretion of the Tke2 effector. (**a**) The growth of *E. coli* K12 cells harboring the pTke2-CT and pTki2 plasmids containing the C-terminal toxin domain of the *tke2* effector and the *tki2* immunity genes, respectively, was determined by measuring the OD at 600 nm. At time zero, either 1 mM isopropyl β-D-1-thiogalactopyranoside (IPTG) and/or 0.02% (w/v) arabinose were added to the LB medium, to induce expression of the *tke2-CT* or/and *tki2* genes, respectively. (**b**) Western blot analyses using an anti-V5 or anti-HA monoclonal antibody to detect the Tke2-CT-V5 or Tki2-HA-tagged proteins. Proteins were prepared from *E. coli* K12 cells grown during 10 h in presence (+) or absence (−) of 1 mM IPTG and/or 0.02% (w/v) arabinose. (**c**) The indicated *P. putida* KT2440 strains bearing a *tke2*-V5-tagged gene were grown in tryptone soya broth (TSB) medium for 5 h. Tke2-V5 was detected in the whole cell and supernatant fractions using a monoclonal anti-V5 antibody. Detection of the β-subunit of the RNA polymerase (β-RNAP) was used as control. The position of the molecular size marker (in kDa) is indicated.

We have shown that the K1 system is functional, and that the corresponding gene cluster encodes several EI pairs including Tke2/Tki2 (Figures 1c, 4a and 5a, and Supplementary Table S4). To assess the functionality of this EI pair, the *tke2* and *tki2* genes were cloned into compatible plasmids and transformed into *E. coli* K12. Expression was induced by the addition of isopropyl β-D-1-thiogalactopyranoside (*tke2*) or arabinose (*tki2*). On induction of the effector gene *tke2*, *E. coli* growth was significantly impaired (Figures 6a and b) but growth could readily be rescued on co-expression of the putative Tki2 immunity protein (Figures 6a and b). This suggests that Tke2/Tki2 is a genuine EI pair.

We assessed whether Tke2 is secreted in a K1-T6SS-dependent manner. The corresponding gene was replaced on the KT2440 chromosome with a version encoding a C-terminally V5-tagged protein. However, Tke2-V5 production was only weakly detected when using this strain (Supplementary Figure S3). In contrast to bacterial killing, which is a highly sensitive assay, detection of secreted T6SS toxins by western blot may need higher level of T6SS expression (Cianfanelli *et al.*, 2016). It has been described in other bacteria that several global regulators are involved in T6SS expression, including the alternative sigma factor RpoN (Bernard *et al.*, 2010, 2011; Sana *et al.*, 2013). The *tke2-V5* chimeric gene was introduced into an *rpoN* mutant and in this strain Tke2 production was considerably increased as compared with the wild-type *P. putida* (Supplementary Figure S3). We thus used this genetic background to analyze Tke2 secretion. Tke2 was produced in both the *rpoN* strain and the isogenic T6SS mutant (*rpoN*Δ*tssA1*), but was only found in the supernatant of the strain with an intact T6SS (Figure 6c). Our results show that Tke2 is an effector of the K1-T6SS and its activity is antagonized by the Tki2 immunity protein.

### *P. putida* outcompetes plant pathogens in a T6SS-dependent manner

**Figure 7:**
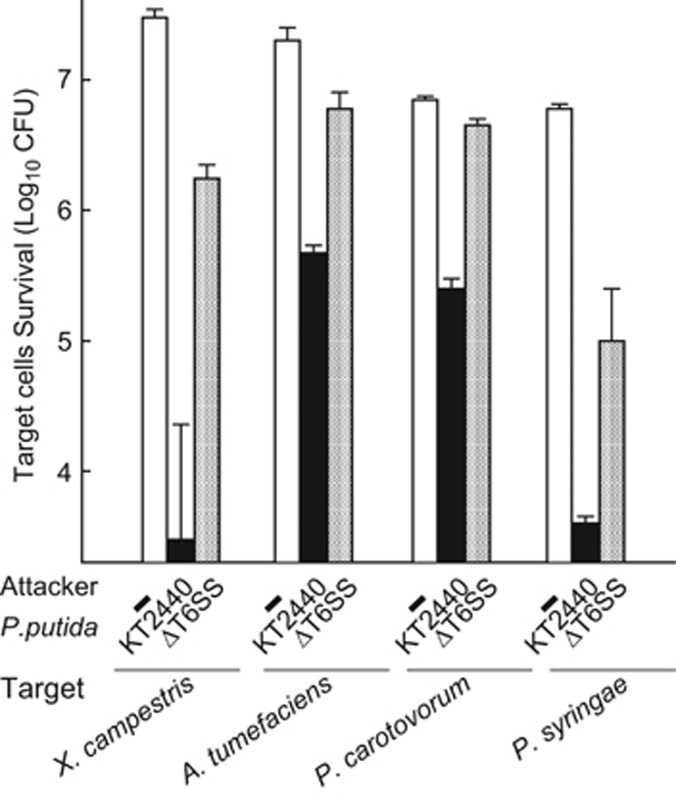
Bactericidal activity of *P. putida* KT2440 against a panel of phytopathogens. *X. campestris*, *A. tumefaciens*, *P. carotovorum* and *P. syringae pv. tomato* strains harbor the pRL662-*gfp* plasmid that confers gentamycin resistance. The *P. putida* KT2440 wild type (WT) and its isogenic Δ*tssA1*Δ*tssM2*Δ*tssM3* triple mutant (ΔT6SS) were co-incubated with the phytopathogens for 24 h. Colony-forming unit (CFU) quantifications were performed on gentamycin selection. The average±s.d. from at least three biological replicates is plotted.

*P. putida* is an efficient biocontrol agent (Amer and Utkhede, 2000; Validov *et al.*, 2007) and we hypothesized that it uses the T6SS to kill ecologically relevant competitors. To test this we selected four plant pathogens, *P. syringae*, *X. campestris*, *Pectobacterium carotovorum* and *Agrobacterium tumefaciens*, which are leading causes of deadly diseases in several economically important crops (Mansfield *et al.*, 2012). The various T6SSs are likely to be differentially expressed *in vitro*, *in vivo*, *in planta* or in the presence of different competitors (Ma *et al.*, 2014). To assess whether the T6SS in general is required for outcompeting plant pathogens and thus involve in plant protection we used a triple T6SS mutant (Δ*tssA1*Δ*tssM2*Δ*tssM3*, also named ΔT6SS), so that none of the K1, K2 or K3 system is at play. First, a competition assay between KT2440 or the triple mutant and the phytopathogens was performed. The *P. putida* wild-type strain caused a 10-fold decrease in survival of *A. tumefaciens* and *P. caratovorum*, and a 1000-fold decrease in the survival of *X. campestris* and *P. syringae* (Figure 7). The *P. putida* T6SS mutant had barely any impact on the survival of any of these bacteria (Figure 7). Our results indicate that KT2440 outcompetes all challenged phytopathogens in a T6SS-dependent manner and suggest a role for this secretion system in biocontrol.

### T6SS-active *P. putida* protects plants from pathogen's attack

**Figure 8:**
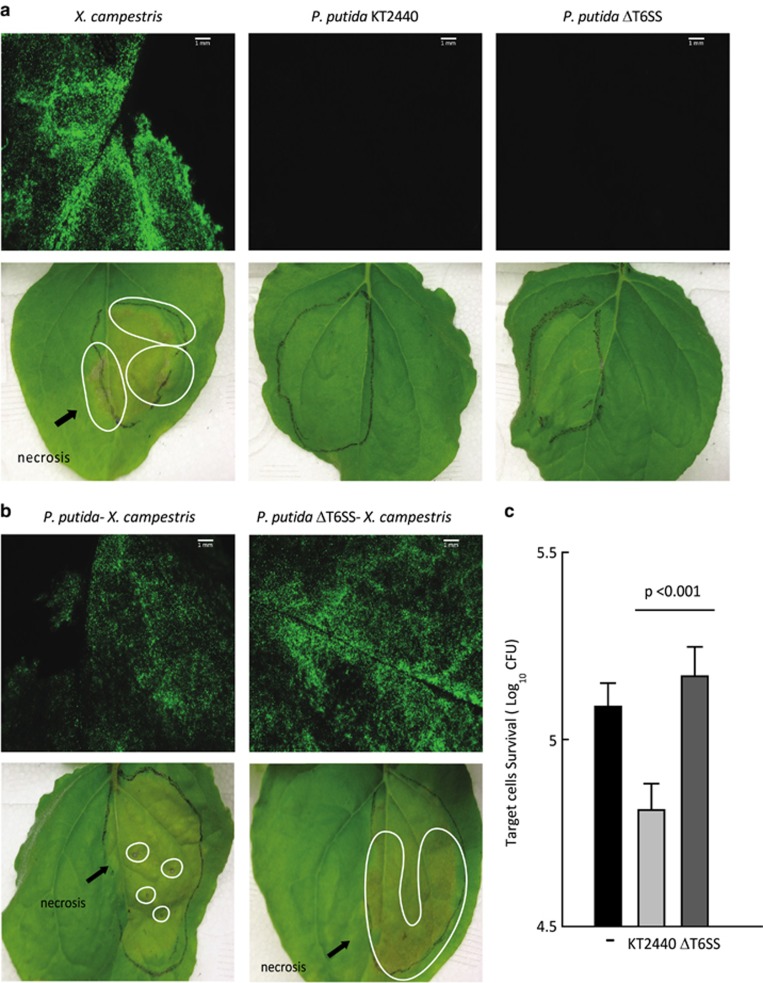
*In planta* competition assay between the biocontrol strain *P. putida* KT2440 and the phytopathogen *X. campestris*. (**a**) Leaves of *N. benthamiana* 24 h (upper panel) and 5 days (lower panel) after being infiltrated with *X. campestris* (pRL662-*gfp*; expressing a plasmid-encoded green fluorescence protein), the *P. putida* KT2440 wild type (WT), or its isogenic Δ*tssA1*Δ*tssM2*Δ*tssM3* triple mutant (ΔT6SS). (**b**) Leaves of *N. benthamiana* 24 h (upper panel) and 5 days (lower panel) after co-infiltration of *X. campestris* (pRL662-*gfp*) with the indicated *P. putida* strain. In upper panel **a** and **b**, the leaves were visualized by fluorescence microscopy using a Leica M205FA stereomicroscope. The necrotic areas resulting from *X. campestris* infection are marked. The deep brown zone of necrosis is spread on a large portion of the leave (right panel), while such spread is far more restricted when the phytopathogen is co-inoculated with a T6SS positive *P. putida* strain (left panel). (**c**) Quantification of *X. campestris* (pRL662-*gfp*) colony-forming units (CFUs) recovered from *N. benthamiana* leaves after 24 h of co-infiltration with the indicated *P. putida* strain. *X. campestris* CFU were quantified after gentamycin (Gm) selection. Graphs represent mean +s.d., of at least five biological replicates with two technical replicates per experiment, statistical significance is indicated *t*-test *P*<0.001.

To assess the ability of *P. putida* to kill phytopathogens in an ecologically relevant set-up, we developed an *in planta* competition assay. We selected *X. campestris* as the pathogen and *N. benthamiana* as the plant model. Leaves were co-infected with *X. campestris* and either *P. putida* wild type or the isogenic ΔT6SS mutant. *X. campestris* was tagged with a green fluorescent protein to monitor *in situ* colonization. *X. campestris-*induced halos of necrosis on the leaves were observed 5 days post infection, whereas inoculation with *P. putida* resulted in healthy-looking leaves (Figure 8a). Remarkably, co-infiltration of *X. campestris* and *P. putida* wild-type strain considerably reduced the necrotic areas produced by *X. campestris* (circled in Figure 8b lower panel). This is not observable with the *P. putida* ΔT6SS mutant and we concluded that interference with *X. campestris* colonization is T6SS dependent (Figure 8b). The protection conferred by *P. putida* is due to reduced survival of *X. campestris* in the leaves (~2.5-fold reduction), as qualitatively observed by fluorescence microscopy (Figure 8b upper panel) and quantitatively measured by colony-forming unit counting (Figure 8c). Our results show that *P. putida* outcompetes *X. campestris* during plant colonization and this process involves the bactericidal properties of the T6SS.

## Discussion

The T6SS was discovered in the bacterial pathogens *V. cholerae* (Pukatzki *et al.*, 2006) and *P. aeruginosa* (Mougous *et al.*, 2006). Since then, an increasing number of studies has provided details on the function and structure of this original bacterial secretion system (Russell *et al.*, 2014; Zoued *et al.*, 2014; Cianfanelli, *et al.*, 2016; Hachani *et al.*, 2016). However, although the presence of the T6SS in non-pathogenic strains is evident (Boyer *et al.*, 2009), little work has been done to understand its relevance in this category of bacteria (Bladergroen *et al.*, 2003; Mougous *et al.*, 2006; Pukatzki *et al.*, 2006; Aschtgen *et al.*, 2008).

### Phylogeny and genetic structure of the *P. putida* T6SS clusters

In this study we have identified a total of 66 T6SS clusters among *P. putida* strains, which suggests that this secretion machine has an important role in *P. putida* physiology and fitness. The *P. putida* T6SS clusters clade within three phylogenetic groups, group 1.2, 2 or 4B (Figure 2). Remarkably, *P. putida* is the only *Pseudomonas* species encoding T6SSs from group 1.2, whereas T6SSs from group 4B are only present in *P. putida* and *P. syringae* (Barret *et al.*, 2011). The *P. putida* KT2440 strain contains two clusters from group 1.2 (K2 and K3) and one cluster from group 4B (K1). The K2-T6SS cluster contains two *orf*s, *vgrG2* and *tssC2*, which present premature stop codons (Supplementary Table S5), implying that this system is not functional. Prematurely interrupted T6SS genes have been identified in functional T6SSs of *Citrobacter rodentium* and *Yersinia pseudotuberculosis* (Gueguen *et al.*, 2014). In these cases, a transcriptional frameshifting caused by a poly-A tract allows the production of functional TssM variants (Gueguen *et al.*, 2014). However, this is unlikely to be the case in KT2440, as poly-A tracts are not found either in *tssC2* or in *vgrG2*. Alternatively, related VgrGs (that is, VgrG3, VgrG4 and VgrG5; Supplementary Figure S4) and TssC proteins (that is, TssC3; Supplementary Table S5) could be shared between different T6SSs.

The K2 and K3 clusters do not encode a ClpV protein, the ATPase responsible for disassembling the T6SS sheath. Yet, orphan *clpV* genes can be used. There are three Clp ATPase-encoding genes in the KT2440 genome (that is, *PP0625*, *PP3316* and *PP4008*), but none encodes a protein from the ClpV family (Supplementary Figure S5). They are ClpA and ClpB members, which are phylogenetically distant from ClpV (Schlieker *et al.*, 2005). Alternatively, the ClpV1 component within the K1 cluster could be shared between the systems but possibly a ClpV component may not be necessary for the function of the group 1.2 T6SS, as some *P. putida* strains (that is, S12, B001, SJ3 and S610) exclusively contain a group 1.2 cluster (Supplementary Table S3). In fact, functional T6SSs lacking the *clpV* gene have been identified in other bacteria (Chow and Mazmanian, 2010; Bröms *et al.*, 2012). Furthermore, the *clpV* gene of *V. cholerae* can be deleted without a total loss of T6SS function (Zheng *et al.*, 2011). After all, other nanomachines structurally comparable to the T6SS such as the contractile-tailed phages or R-type pyocins, do not use a ClpV homolog for their function. Instead, recently discovered phage-like protein translocation structures are encoded within gene clusters that also carry a *clpV* homolog (Kube and Wendler, 2015). This type of structure may have evolved divergently with some of the T6SS subgroups and acquired ClpV from ancestral systems.

### Antibacterial activity of the *P. putida* KT2440 T6SS

The main role of the T6SS is to inject effectors into eukaryotic or prokaryotic prey cells (Alcoforado Diniz *et al.*, 2015; Hachani *et al.*, 2016). We identified an impressive battery of 10 potential T6SS effectors in *P. putida* KT2440. This is not unique but suggests that *P. putida* is primed to fight a wide range of competing organisms. At least three EI pairs are encoded within the K1-T6SS cluster (that is, *tke1-tki1*, *tke2-tki2* and *tke3-tki3*), which belongs to the uncharacterized plant-related group (group 4B, Figure 2). A remarkable characteristic of the system is the presence of a conserved accessory gene, *tagX*, systematically absent from other T6SS groups and which is a hallmark for group 4B systems. Here we show that suitable preys for the K1-T6SS are bacterial cells, and that the Tke2 toxin contributes to the antibacterial activity. Tke2 contains a canonical Rhs-effector domain organization, which includes an N-terminal PAAR motif, a central domain of conserved Rhs-repeats and a C-terminal toxic domain. Although the function of the Rhs domain is still unknown, it has been suggested that it forms a shell structure that encapsulates the C-terminal region of effectors (Busby *et al.*, 2013; Supplementary Figure S6). Furthermore, a specific adaptor named EagR (after ‘effector-associated gene') that contains the DUF1795 domain, has been involved in the secretion of PAAR/Rhs effectors (Alcoforado Diniz and Coulthurst, 2015). Two different proteins containing DUF1795 domains are encoded immediately upstream *tke2* (*eagR1a* and *eagR1b*, Figure 1c). Although the function of these adaptors has not been analyzed yet, it is possible that both function together to assist Tke2 secretion. The recurrent association between PAAR/Rhs effectors and EagR adaptors is furthermore confirmed by the association of *tke4*, encoding another *P. putida* PAAR/Rhs effector (Figures 1c and 4a, and Supplementary Figure S6), with an *eagR* gene (*eagR2*).

### Biocontrol properties of the *P. putida* T6SS

It is becoming increasingly obvious that the antimicrobial properties of the T6SS could be instrumental for the control of polymicrobial populations in excluding foes from natural and ecologically relevant environments. For instance, a clear correlation between activation of T6SS, enhanced fitness and subsequent antagonism against other bacteria has been observed with *Vibrio parahaemolyticus* in marine niches (Salomon *et al.*, 2013). This suggested that T6SSs are key for survival and persistence of specialized species in specific habitats. In the lungs of cystic fibrosis patients, *P. aeruginosa* can persist for years, while the diversity of species that primarily colonizes this environment decreases over time (Marshall *et al.*, 2015). *P. aeruginosa* isolates from cystic fibrosis patients have highly active T6SSs (Mougous *et al.*, 2006; Moscoso *et al.*, 2011), which suggests that T6SSs contribute to the colonization advantage of *P. aeruginosa* over other species. In agreement with these observations, the T6SS has been proposed to be crucial in the establishment/evolution of the gut microbiome (Russell, *et al.*, 2014; Cianfanelli, *et al.*, 2016). Half of the human-associated Bacteroidetes, the dominant phyla in the human gut, not only encode T6SSs (Coyne *et al.*, 2016) and possess a wide range of T6SS effectors (Chatzidaki-Livanis *et al.*, 2016) but accumulate immunity genes against other T6SS effectors (Wexler *et al.*, 2016). This strongly supports that T6SS is a selective mechanism involved in the establishment of gut communities. These remarkable properties of the T6SS are obviously useful in the development of biocontrol strains. The T6SS was originally discovered in *Rhizobium leguminosarum* and involved in pea nodulation (Bladergroen *et al.*, 2003), but barely any studies have demonstrated the potential that such system may have in the context of the plant microbiome. A parallel can be made between the gut and the rhizosphere, as both are eukaryotic-based environments hosting a symbiotic relationship with a complex microbial community (Stone, 2016). Both animals and plants depend on their microbiome to protect themselves against pathogens and to help assimilate necessary nutrients (Carmody *et al.*, 2015; Haney and Ausubel, 2015; Haney *et al.*, 2015). As a defence strategy, many plant species promote the development of a specific microbiome in the rhizosphere, which has antagonistic activity against soil-borne pathogens (Cook *et al.*, 1995; Weller *et al.*, 2002; Lebeis *et al.*, 2015). Although the mechanisms for pathogen suppression are not completely understood, they include the production of bioactive metabolites such as antibiotics, bacteriocins and siderophores (Weller, 2007). However, these mechanisms fail to account for the full level of protection conferred by the biocontrol organism (Matilla *et al.*, 2010). Here we report for the first time that the T6SS might be a primary mechanism for phytopathogen control. Indeed, we demonstrate that the crop protection agent *P. putida* KT2440 readily outcompetes a panel of economically important phytopathogens and that the efficient destruction of the pest is mostly T6SS dependent. This property can likely be transferred to the field, as this effect was observed *in vitro* but also *in vivo* by demonstrating that *P. putida* protects plant leaves from the deleterious effect of *X. campestris*.

In our study we have used a laboratory setup and further trials in crop plants are needed so that in-depth investigation of the impact of KT2440 in the rhizosphere can be assessed. Nevertheless, our finding shows that the T6SS can be used by environmental strains to protect plants from the attack of bacterial pathogens and can thus be considered as a plant health warden. This opens new possibilities in the selection of biocontrol agents used for biotechnological applications. Noticeably, the poor specificity of the T6SS (Hood *et al.*, 2010) may allow such biocontrol organism to also fight eukaryotic pathogens belonging to different kingdoms including nematodes and fungi.

## References

[bib1] Alcoforado Diniz J, Coulthurst SJ. (2015). Intraspecies competition in serratia marcescens is mediated by type VI-secreted Rhs effectors and a conserved effector-associated accessory protein. J Bacteriol 197: 2350–2360. 25939831 10.1128/JB.00199-15 PMC4524185

[bib2] Alcoforado Diniz J, Liu Y-CC, Coulthurst SJ. (2015). Molecular weaponry: diverse effectors delivered by the Type VI secretion system. Cell Microbiol 17: 1742–1751. 26432982 10.1111/cmi.12532 PMC4832377

[bib3] Amer GA, Utkhede RS. (2000). Development of formulations of biological agents for management of root rot of lettuce and cucumber. Can J Microbiol 46: 809–816. 11006841 10.1139/w00-063

[bib4] Aschtgen M-SS, Bernard CS, De Bentzmann S, Lloubès R, Cascales E. (2008). SciN is an outer membrane lipoprotein required for type VI secretion in enteroaggregative *Escherichia coli*. J Bacteriol 190: 7523–7531. 18805985 10.1128/JB.00945-08 PMC2576670

[bib5] Aschtgen M-SS, Thomas MS, Cascales E. (2010). Anchoring the type VI secretion system to the peptidoglycan: TssL, TagL, TagP... what else? Virulence 1: 535–540. 21178498 10.4161/viru.1.6.13732

[bib6] Barret M, Egan F, Fargier E, Morrissey JP, O'Gara F. (2011). Genomic analysis of the type VI secretion systems in *Pseudomonas* spp.: novel clusters and putative effectors uncovered. Microbiology 157: 1726–1739. 21474537 10.1099/mic.0.048645-0

[bib7] Basler M, Ho BT, Mekalanos JJ. (2013). Tit-for-tat: type VI secretion system counterattack during bacterial cell-cell interactions. Cell 152: 884–894. 23415234 10.1016/j.cell.2013.01.042 PMC3616380

[bib8] Basler M, Mekalanos JJ. (2012). Type 6 secretion dynamics within and between bacterial cells. Science 337: 815. 22767897 10.1126/science.1222901 PMC3557511

[bib9] Bernard CS, Brunet YR, Gavioli M, Lloubès R, Cascales E. (2011). Regulation of type VI secretion gene clusters by sigma54 and cognate enhancer binding proteins. J Bacteriol 193: 2158–2167. 21378190 10.1128/JB.00029-11 PMC3133059

[bib10] Bernard CS, Brunet YR, Gueguen E, Cascales E. (2010). Nooks and crannies in type VI secretion regulation. J Bacteriol 192: 3850–3860. 20511495 10.1128/JB.00370-10 PMC2916374

[bib11] Bladergroen MR, Badelt K, Spaink HP. (2003). Infection-blocking genes of a symbiotic *Rhizobium leguminosarum* strain that are involved in temperature-dependent protein secretion. Mol Plant Microbe Interact 16: 53–64. 12580282 10.1094/MPMI.2003.16.1.53

[bib12] Boratyn GM, Camacho C, Cooper PS, Coulouris G, Fong A, Ma N et al. (2013). BLAST: a more efficient report with usability improvements. Nucleic Acids Res 41: W29–W33. 23609542 10.1093/nar/gkt282 PMC3692093

[bib13] Boyer F, Fichant G, Berthod J, Vandenbrouck Y, Attree I. (2009). Dissecting the bacterial type VI secretion system by a genome wide *in silico* analysis: what can be learned from available microbial genomic resources? BMC Genomics 10: 104. 19284603 10.1186/1471-2164-10-104 PMC2660368

[bib14] Bröms JE, Meyer L, Sun K, Lavander M, Sjöstedt A. (2012). Unique substrates secreted by the type VI secretion system of *Francisella tularensis* during intramacrophage infection. PLoS ONE 7: e50473. 23185631 10.1371/journal.pone.0050473 PMC3502320

[bib15] Burtnick MN, Brett PJ, Harding SV, Ngugi SA, Ribot WJ, Chantratita N et al. (2011). The cluster 1 type VI secretion system is a major virulence determinant in *Burkholderia pseudomallei*. Infect Immun 79: 1512–1525. 21300775 10.1128/IAI.01218-10 PMC3067527

[bib16] Busby JN, Panjikar S, Landsberg MJ, Hurst MR, Lott JS. (2013). The BC component of ABC toxins is an RHS-repeat-containing protein encapsulation device. Nature 501: 547–550. 23913273 10.1038/nature12465

[bib17] Carmody RN, Gerber GK, Luevano JM, Gatti DM, Somes L, Svenson KL et al. (2015). Diet dominates host genotype in shaping the murine gut microbiota. Cell Host Microbe 17: 72–84. 25532804 10.1016/j.chom.2014.11.010 PMC4297240

[bib18] Cascales E, Cambillau C. (2012). Structural biology of type VI secretion systems. Philos Trans R Soc Lond B Biol Sci 367: 1102–1111. 22411981 10.1098/rstb.2011.0209 PMC3297440

[bib19] Chatzidaki-Livanis M, Geva-Zatorsky N, Comstock LE. (2016). *Bacteroides fragilis* type VI secretion systems use novel effector and immunity proteins to antagonize human gut Bacteroidales species. Proc Natl Acad Sci USA 113: 3627–3632. 26951680 10.1073/pnas.1522510113 PMC4822612

[bib20] Choi K-HH, Kumar A, Schweizer HP. (2006). A 10-min method for preparation of highly electrocompetent *Pseudomonas aeruginosa* cells: application for DNA fragment transfer between chromosomes and plasmid transformation. J Microbiol Methods 64: 391–397. 15987659 10.1016/j.mimet.2005.06.001

[bib21] Chow J, Mazmanian SK. (2010). A pathobiont of the microbiota balances host colonization and intestinal inflammation. Cell Host Microbe 7: 265–276. 20413095 10.1016/j.chom.2010.03.004 PMC2859213

[bib22] Cianfanelli FR, Alcoforado Diniz J, Guo M, De Cesare V, Trost M, Coulthurst SJ. (2016). VgrG and PAAR proteins define distinct versions of a functional type VI secretion system. PLoS Pathog 12: e1005735. 27352036 10.1371/journal.ppat.1005735 PMC4924876

[bib23] Cianfanelli FR, Monlezun L, Coulthurst SJ. (2016). Aim, load, fire: the type VI secretion system, a bacterial nanoweapon. Trends Microbiol 24: 51–62. 26549582 10.1016/j.tim.2015.10.005

[bib24] Cook RJ, Thomashow LS, Weller DM, Fujimoto D, Mazzola M, Bangera G et al. (1995). Molecular mechanisms of defense by rhizobacteria against root disease. Proc Natl Acad Sci USA 92: 4197–4201. 11607544 10.1073/pnas.92.10.4197 PMC41910

[bib25] Coyne MJ, Roelofs KG, Comstock LE. (2016). Type VI secretion systems of human gut Bacteroidales segregate into three genetic architectures, two of which are contained on mobile genetic elements. BMC Genomics 17: 58. 26768901 10.1186/s12864-016-2377-z PMC4714493

[bib26] de Pace F, Nakazato G, Pacheco A, de Paiva JB, Sperandio V, da Silveira WD. (2010). The type VI secretion system plays a role in type 1 fimbria expression and pathogenesis of an avian pathogenic *Escherichia coli* strain. Infect Immun 78: 4990–4998. 20855516 10.1128/IAI.00531-10 PMC2981326

[bib27] Dong TG, Ho BT, Yoder-Himes DR, Mekalanos JJ. (2013). Identification of T6SS-dependent effector and immunity proteins by Tn-seq in *Vibrio cholerae*. Proc Natl Acad Sci USA 110: 2623–2628. 23362380 10.1073/pnas.1222783110 PMC3574944

[bib28] Durand E, Cambillau C, Cascales E, Journet L. (2014). VgrG, Tae, Tle, and beyond: the versatile arsenal of type VI secretion effectors. Trends Microbiol 22: 498–507. 25042941 10.1016/j.tim.2014.06.004

[bib29] Durand E, Nguyen VS, Zoued A, Logger L, Péhau-Arnaudet G, Aschtgen M-SS et al. (2015). Biogenesis and structure of a type VI secretion membrane core complex. Nature 523: 555–560. 26200339 10.1038/nature14667

[bib30] Espinosa-Urgel M, Salido A, Ramos JL. (2000). Genetic analysis of functions involved in adhesion of *Pseudomonas putida* to seeds. J Bacteriol 182: 2363–2369. 10762233 10.1128/jb.182.9.2363-2369.2000 PMC111295

[bib31] Filloux A. (2011). Protein secretion systems in *Pseudomonas aeruginosa*: an essay on diversity, evolution, and function. Front Microbiol 2: 155. 21811488 10.3389/fmicb.2011.00155 PMC3140646

[bib32] Filloux A, Freemont P. (2016). Structural biology: baseplates in contractile machines. Nat Microbiol 1: 16104. 27572982 10.1038/nmicrobiol.2016.104

[bib33] Finn RD, Coggill P, Eberhardt RY, Eddy SR, Mistry J, Mitchell AL et al. (2016). The Pfam protein families database: towards a more sustainable future. Nucleic Acids Res 44: D279–D285. 26673716 10.1093/nar/gkv1344 PMC4702930

[bib34] Gomi M, Sonoyama M, Mitaku S. (2004). High performance system for signal peptide prediction: SOSUIsignal. Chem Bio Info J 4: 142–147.

[bib35] Gotfredsen M, Gerdes K. (1998). The *Escherichia coli* relBE genes belong to a new toxin-antitoxin gene family. Mol Microbiol 29: 1065–1076. 9767574 10.1046/j.1365-2958.1998.00993.x

[bib36] Gueguen E, Wills NM, Atkins JF, Cascales E. (2014). Transcriptional frameshifting rescues *Citrobacter rodentium* type VI secretion by the production of two length variants from the prematurely interrupted tssM gene. PLoS Genet 10: e1004869. 25474156 10.1371/journal.pgen.1004869 PMC4256274

[bib37] Guzman LM, Belin D, Carson MJ, Beckwith J. (1995). Tight regulation, modulation, and high-level expression by vectors containing the arabinose PBAD promoter. J Bacteriol 177: 4121–4130. 7608087 10.1128/jb.177.14.4121-4130.1995 PMC177145

[bib38] Hachani A, Allsopp LP, Oduko Y, Filloux A. (2014). The VgrG proteins are ‘A la carte' delivery systems for bacterial type VI effectors. J Biol Chem 289: 17872–17884. 24794869 10.1074/jbc.M114.563429 PMC4067218

[bib39] Hachani A, Lossi N, Hamilton A, Jones C, Bleves S, Albesa-Jové D et al. (2011). Type VI secretion system in *Pseudomonas aeruginosa* secretion and multimerization of VgrG proteins. J Biol Chem 286: 12317–12327. 21325275 10.1074/jbc.M110.193045 PMC3069435

[bib40] Hachani A, Lossi NS, Filloux A. (2013). A visual assay to monitor T6SS-mediated bacterial competition. J Vis Exp 73: e50103. 10.3791/50103 PMC3639552 23542679

[bib41] Hachani A, Wood TE, Filloux A. (2016). Type VI secretion and anti-host effectors. Curr Opin Microbiol 29: 81–93. 26722980 10.1016/j.mib.2015.11.006

[bib42] Haney CH, Ausubel FM. (2015). MICROBIOME. Plant microbiome blueprints. Science 349: 788–789. 26293938 10.1126/science.aad0092

[bib43] Haney CH, Samuel BS, Bush J, Ausubel FM. (2015). Associations with rhizosphere bacteria can confer an adaptive advantage to plants. Nat Plants 1: 15051. 27019743 10.1038/nplants.2015.51 PMC4806546

[bib44] Ho B, Dong T, Mekalanos J. (2013). A view to a kill: the bacterial type VI secretion system. Cell Host Microbe 15: 9–21. 24332978 10.1016/j.chom.2013.11.008 PMC3936019

[bib45] Hoang TT, Kutchma AJ, Becher A, Schweizer HP. (2000). Integration-proficient plasmids for *Pseudomonas aeruginosa*: site-specific integration and use for engineering of reporter and expression strains. Plasmid 43: 59–72. 10610820 10.1006/plas.1999.1441

[bib46] Hood R, Singh P, Hsu F, Güvener T, Carl M, Trinidad R et al. (2010). A type VI secretion system of *Pseudomonas aeruginosa* targets a toxin to bacteria. Cell Host Microbe 7: 25–37. 20114026 10.1016/j.chom.2009.12.007 PMC2831478

[bib47] Huang H, Yuan HS. (2007). The conserved asparagine in the HNH motif serves an important structural role in metal finger endonucleases. J Mol Biol 368: 812–821. 17368670 10.1016/j.jmb.2007.02.044

[bib48] Imai K, Asakawa N, Tsuji T, Akazawa F, Ino A, Sonoyama M et al. (2008). SOSUI-GramN: high performance prediction for sub-cellular localization of proteins in gram-negative bacteria. Bioinformation 2: 417–421. 18795116 10.6026/97320630002417 PMC2533062

[bib49] Kaniga K, Delor I, Cornelis GR. (1991). A wide-host-range suicide vector for improving reverse genetics in gram-negative bacteria: inactivation of the blaA gene of *Yersinia enterocolitica*. Gene 109: 137–141. 1756974 10.1016/0378-1119(91)90599-7

[bib50] Kapitein N, Bönemann G, Pietrosiuk A, Seyffer F, Hausser I, Locker JK et al. (2013). ClpV recycles VipA/VipB tubules and prevents non-productive tubule formation to ensure efficient type VI protein secretion. Mol Microbiol 87: 1013–1028. 23289512 10.1111/mmi.12147

[bib51] Katzen F, Ferreiro DU, Oddo CG, Ielmini MV, Becker A, Pühler A et al. (1998). *Xanthomonas campestris* pv. campestris gum mutants: effects on xanthan biosynthesis and plant virulence. J Bacteriol 180: 1607–1617. 9537354 10.1128/jb.180.7.1607-1617.1998 PMC107069

[bib52] Kelley LA, Mezulis S, Yates CM, Wass MN, Sternberg MJ. (2015). The Phyre2 web portal for protein modeling, prediction and analysis. Nat Protoc 10: 845–858. 25950237 10.1038/nprot.2015.053 PMC5298202

[bib53] Krogh A, Larsson B, von Heijne G, Sonnhammer EL. (2001). Predicting transmembrane protein topology with a hidden Markov model: application to complete genomes. J Mol Biol 305: 567–580. 11152613 10.1006/jmbi.2000.4315

[bib54] Kube S, Kapitein N, Zimniak T, Herzog F, Mogk A, Wendler P. (2014). Structure of the VipA/B type VI secretion complex suggests a contraction-state-specific recycling mechanism. Cell Rep 8: 20–30. 24953649 10.1016/j.celrep.2014.05.034

[bib55] Kube S, Wendler P. (2015). Structural comparison of contractile nanomachines. AIMS Biophys 2: 88–115.

[bib56] Kudryashev M, Wang RY, Brackmann M, Scherer S, Maier T, Baker D et al. (2015). Structure of the type VI secretion system contractile sheath. Cell 160: 952–962. 25723169 10.1016/j.cell.2015.01.037 PMC4359589

[bib57] Lebeis SL, Paredes SH, Lundberg DS, Breakfield N, Gehring J, McDonald M et al. (2015). PLANT MICROBIOME. Salicylic acid modulates colonization of the root microbiome by specific bacterial taxa. Science 349: 860–864. 26184915 10.1126/science.aaa8764

[bib58] Leiman PG, Basler M, Ramagopal UA, Bonanno JB, Sauder JM, Pukatzki S et al. (2009). Type VI secretion apparatus and phage tail-associated protein complexes share a common evolutionary origin. Proc Natl Acad Sci USA 106: 4154–4159. 19251641 10.1073/pnas.0813360106 PMC2657435

[bib59] Letunic I, Doerks T, Bork P. (2015). SMART: recent updates, new developments and status in 2015. Nucleic Acids Res 43: D257–D260. 25300481 10.1093/nar/gku949 PMC4384020

[bib60] Liang X, Moore R, Wilton M, Wong MJ, Lam L, Dong TG. (2015). Identification of divergent type VI secretion effectors using a conserved chaperone domain. Proc Natl Acad Sci USA 112: 9106–9111. 26150500 10.1073/pnas.1505317112 PMC4517263

[bib61] Lin J-SS, Ma L-SS, Lai E-MM. (2013). Systematic dissection of the agrobacterium type VI secretion system reveals machinery and secreted components for subcomplex formation. PLoS One 8: e67647. 23861778 10.1371/journal.pone.0067647 PMC3702570

[bib62] Lyons E, Freeling M. (2008). How to usefully compare homologous plant genes and chromosomes as DNA sequences. Plant J 53: 661–673. 18269575 10.1111/j.1365-313X.2007.03326.x

[bib63] Ma AT, Mekalanos JJ. (2010). *In vivo* actin cross-linking induced by *Vibrio cholerae* type VI secretion system is associated with intestinal inflammation. Proc Natl Acad Sci USA 107: 4365–4370. 20150509 10.1073/pnas.0915156107 PMC2840160

[bib64] Ma L-SS, Hachani A, Lin J-SS, Filloux A, Lai E-MM. (2014). *Agrobacterium tumefaciens* deploys a superfamily of type VI secretion DNase effectors as weapons for interbacterial competition in planta. Cell Host Microbe 16: 94–104. 24981331 10.1016/j.chom.2014.06.002 PMC4096383

[bib65] Mansfield J, Genin S, Magori S, Citovsky V, Sriariyanum M, Ronald P et al. (2012). Top 10 plant pathogenic bacteria in molecular plant pathology. Mol Plant Pathol 13: 614–629. 22672649 10.1111/j.1364-3703.2012.00804.x PMC6638704

[bib66] Marchi M, Boutin M, Gazengel K, Rispe C, Gauthier J-PP, Guillerm-Erckelboudt A-YY et al. (2013). Genomic analysis of the biocontrol strain *Pseudomonas fluorescens* Pf29Arp with evidence of T3SS and T6SS gene expression on plant roots. Environ Microbiol Rep 5: 393–403. 23754720 10.1111/1758-2229.12048

[bib67] Marshall B, Elbert A, Petren K, Rizvi S, Fink A, Ostrenga J et al. (2015) Cystic Fibrosis Foundation Patient Registry 2014 Annual Data Report. 2015 Cystic Fibrosis Foundation: Bethesda, Maryland, USA.

[bib68] Matilla M, Ramos J, Bakker P, Doornbos R, Badri D, Vivanco J et al. (2010). *Pseudomonas putida* KT2440 causes induced systemic resistance and changes in *Arabidopsis* root exudation. Environ Microbiol Rep 2: 381–388. 23766110 10.1111/j.1758-2229.2009.00091.x

[bib69] Miyata ST, Kitaoka M, Brooks TM, McAuley SB, Pukatzki S. (2011). *Vibrio cholerae* requires the type VI secretion system virulence factor VasX to kill *Dictyostelium discoideum*. Infect Immun 79: 2941–2949. 21555399 10.1128/IAI.01266-10 PMC3191968

[bib70] Molina L, Ramos C, Duque E, Ronchel MC, Garcia JM, Wyke L et al. (2000). Survival of *Pseudomonas putida* KT2440 in soil and in the rhizosphere of plants under greenhouse and environmental conditions. Soil Biol Biochem 32: 315–321.

[bib71] Moscoso JA, Mikkelsen H, Heeb S, Williams P, Filloux A. (2011). The *Pseudomonas aeruginosa* sensor RetS switches type III and type VI secretion via c-di-GMP signalling. Environ Microbiol 13: 3128–3138. 21955777 10.1111/j.1462-2920.2011.02595.x

[bib72] Mougous JD, Cuff ME, Raunser S, Shen A, Zhou M, Gifford CA et al. (2006). A virulence locus of *Pseudomonas aeruginosa* encodes a protein secretion apparatus. Science 312: 1526–1530. 16763151 10.1126/science.1128393 PMC2800167

[bib73] Murdoch SL, Trunk K, English G, Fritsch MJ, Pourkarimi E, Coulthurst SJ. (2011). The opportunistic pathogen *Serratia marcescens* utilizes type VI secretion to target bacterial competitors. J Bacteriol 193: 6057–6069. 21890705 10.1128/JB.05671-11 PMC3194891

[bib74] Okonechnikov K, Golosova O, Fursov M. (2012). Unipro UGENE: a unified bioinformatics toolkit. Bioinformatics 28: 1166–1167. 22368248 10.1093/bioinformatics/bts091

[bib75] Petersen TN, Brunak S, von Heijne G, Nielsen H. (2011). SignalP 4.0: discriminating signal peptides from transmembrane regions. Nat Methods 8: 785–786. 21959131 10.1038/nmeth.1701

[bib76] Planamente S, Salih O, Manoli E, Albesa-Jové D, Freemont PS, Filloux A. (2016). TssA forms a gp6-like ring attached to the type VI secretion sheath. EMBO J 35: 1613–1627. 27288401 10.15252/embj.201694024 PMC4969574

[bib77] Pukatzki S, Ma AT, Sturtevant D, Krastins B, Sarracino D, Nelson WC et al. (2006). Identification of a conserved bacterial protein secretion system in *Vibrio cholerae* using the Dictyostelium host model system. Proc Natl Acad Sci USA 103: 1528–1533. 16432199 10.1073/pnas.0510322103 PMC1345711

[bib78] Ramos-Gonzalez MI, Duque E, Ramos JL. (1991). Conjugational transfer of recombinant DNA in cultures and in soils: host range of *Pseudomonas putida* TOL plasmids. Appl Environ Microbiol 57: 3020–3027. 1660698 10.1128/aem.57.10.3020-3027.1991 PMC183914

[bib79] Rosales-Reyes R, Skeldon AM, Aubert DF, Valvano MA. (2012). The type VI secretion system of *Burkholderia cenocepacia* affects multiple Rho family GTPases disrupting the actin cytoskeleton and the assembly of NADPH oxidase complex in macrophages. Cell Microbiol 14: 255–273. 22023353 10.1111/j.1462-5822.2011.01716.x

[bib80] Russell AB, Peterson SB, Mougous JD. (2014). Type VI secretion system effectors: poisons with a purpose. Nat Rev Microbiol 12: 137–148. 24384601 10.1038/nrmicro3185 PMC4256078

[bib81] Russell AB, Wexler AG, Harding BN, Whitney JC, Bohn AJ, Goo YA et al. (2014). A type VI secretion-related pathway in Bacteroidetes mediates interbacterial antagonism. Cell Host Microbe 16: 227–236. 25070807 10.1016/j.chom.2014.07.007 PMC4136423

[bib82] Salomon D, Gonzalez H, Updegraff BL, Orth K. (2013). *Vibrio parahaemolyticus* type VI secretion system 1 is activated in marine conditions to target bacteria, and is differentially regulated from system 2. PLoS One 8: e61086. 23613791 10.1371/journal.pone.0061086 PMC3628861

[bib83] Salomon D, Kinch LN, Trudgian DC, Guo X, Klimko JA, Grishin NV et al. (2014). Marker for type VI secretion system effectors. Proc Natl Acad Sci USA 111: 9271–9276. 24927539 10.1073/pnas.1406110111 PMC4078801

[bib84] Sambrook J, Maniatis T, Fritsch EF. (1989) Molecular Cloning: A Laboratory Manual. Cold Spring Harbor Laboratory: Cold Spring Harbor, NY.

[bib85] Sana TG, Soscia C, Tonglet CMM, Garvis S, Bleves S. (2013). Divergent control of two type VI secretion systems by RpoN in *Pseudomonas aeruginosa*. PLoS One 8: e76030. 24204589 10.1371/journal.pone.0076030 PMC3804575

[bib86] Schlieker C, Zentgraf H, Dersch P, Mogk A. (2005). ClpV, a unique Hsp100/Clp member of pathogenic proteobacteria. Biol Chem 386: 1115–1127. 16307477 10.1515/BC.2005.128

[bib87] Shneider MM, Buth SA, Ho BT, Basler M, Mekalanos JJ, Leiman PG. (2013). PAAR-repeat proteins sharpen and diversify the type VI secretion system spike. Nature 500: 350–353. 23925114 10.1038/nature12453 PMC3792578

[bib88] Silverman JM, Agnello DM, Zheng H, Andrews BT, Li M, Catalano CE et al. (2013). Haemolysin coregulated protein is an exported receptor and chaperone of type VI secretion substrates. Mol Cell 51: 584–593. 23954347 10.1016/j.molcel.2013.07.025 PMC3844553

[bib89] Silverman JM, Austin LS, Hsu F, Hicks KG, Hood RD, Mougous JD. (2011). Separate inputs modulate phosphorylation-dependent and -independent type VI secretion activation. Mol Microbiol 82: 1277–1290. 22017253 10.1111/j.1365-2958.2011.07889.x PMC3590308

[bib90] Stone M. (2016). Root and gut microbiomes are strikingly similar. Microbe 11: 107–110.

[bib91] Suarez G, Sierra JC, Sha J, Wang S, Erova TE, Fadl AA et al. (2008). Molecular characterization of a functional type VI secretion system from a clinical isolate of *Aeromonas hydrophila*. Microb Pathog 44: 344–361. 18037263 10.1016/j.micpath.2007.10.005 PMC2430056

[bib92] Tamura K, Stecher G, Peterson D, Filipski A, Kumar S. (2013). MEGA6: molecular evolutionary genetics analysis version 6.0. Mol Biol Evol 30: 2725–2729. 24132122 10.1093/molbev/mst197 PMC3840312

[bib93] Unterweger D, Kostiuk B, Ötjengerdes R, Wilton A, Diaz-Satizabal L, Pukatzki S. (2015). Chimeric adaptor proteins translocate diverse type VI secretion system effectors in *Vibrio cholerae*. EMBO J 34: 2198–2210. 26194724 10.15252/embj.201591163 PMC4557670

[bib94] Validov S, Kamilova F, Qi S, Stephan D, Wang JJ, Makarova N et al. (2007). Selection of bacteria able to control *Fusarium oxysporum* f. sp. *radicis-lycopersici* in stonewool substrate. J Appl Microbiol 102: 461–471. 17241352 10.1111/j.1365-2672.2006.03083.x

[bib95] Vasseur P, Vallet-Gely I, Soscia C, Genin S, Filloux A. (2005). The pel genes of the *Pseudomonas aeruginosa* PAK strain are involved at early and late stages of biofilm formation. Microbiology 151: 985–997. 15758243 10.1099/mic.0.27410-0

[bib96] Weinert C, Morger D, Djekic A, Grütter MG, Mittl PR. (2015). Crystal structure of TRIM20 C-terminal coiled-coil/B30.2 fragment: implications for the recognition of higher order oligomers. Sci Rep 5: 10819. 26043233 10.1038/srep10819 PMC4455283

[bib97] Weller DM. (2007). *Pseudomonas* biocontrol agents of soilborne pathogens: looking back over 30 years. Phytopathology 97: 250–256. 18944383 10.1094/PHYTO-97-2-0250

[bib98] Weller DM, Raaijmakers JM, Gardener BB, Thomashow LS. (2002). Microbial populations responsible for specific soil suppressiveness to plant pathogens. Annu Rev Phytopathol 40: 309–348. 12147763 10.1146/annurev.phyto.40.030402.110010

[bib99] Wexler AG, Bao Y, Whitney JC, Bobay L-MM, Xavier JB, Schofield WB et al. (2016). Human symbionts inject and neutralize antibacterial toxins to persist in the gut. Proc Natl Acad Sci USA 113: 3639–3644. 26957597 10.1073/pnas.1525637113 PMC4822603

[bib100] Whitney JC, Beck CM, Goo YA, Russell AB, Harding BN, De Leon JA et al. (2014). Genetically distinct pathways guide effector export through the type VI secretion system. Mol Microbiol 92: 529–542. 24589350 10.1111/mmi.12571 PMC4049467

[bib101] Whitney JC, Quentin D, Sawai S, LeRoux M, Harding BN, Ledvina HE et al. (2015). An interbacterial NAD(P)(+) glycohydrolase toxin requires elongation factor Tu for delivery to target cells. Cell 163: 607–619. 26456113 10.1016/j.cell.2015.09.027 PMC4624332

[bib102] Winsor GL, Griffiths EJ, Lo R, Dhillon BK, Shay JA, Brinkman FS. (2016). Enhanced annotations and features for comparing thousands of *Pseudomonas* genomes in the Pseudomonas genome database. Nucleic Acids Res 44: D646–D653. 26578582 10.1093/nar/gkv1227 PMC4702867

[bib103] Yu NY, Wagner JR, Laird MR, Melli G, Rey S, Lo R et al. (2010). PSORTb 3.0: improved protein subcellular localization prediction with refined localization subcategories and predictive capabilities for all prokaryotes. Bioinformatics 26: 1608–1615. 20472543 10.1093/bioinformatics/btq249 PMC2887053

[bib104] Zheng J, Ho B, Mekalanos JJ. (2011). Genetic analysis of anti-amoebae and anti-bacterial activities of the type VI secretion system in *Vibrio cholerae*. PLoS One 6: e23876. 21909372 10.1371/journal.pone.0023876 PMC3166118

[bib105] Zoued A, Brunet YR, Durand E, Aschtgen M-SS, Logger L, Douzi B et al. (2014). Architecture and assembly of the Type VI secretion system. Biochim Biophys Acta 1843: 1664–1673. 24681160 10.1016/j.bbamcr.2014.03.018

