# Supplementary figures and images for: The Pseudomonas putida T6SS is a plant warden against phytopathogens
Source: ISME J. 2017 Jan 3;11(4):972–87. doi: 10.1038/ismej.2016.169 (PMC5363822; doi:10.1038/ismej.2016.169)

Figure S1

Group 1.2

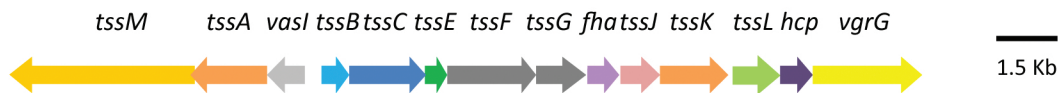

Group 2

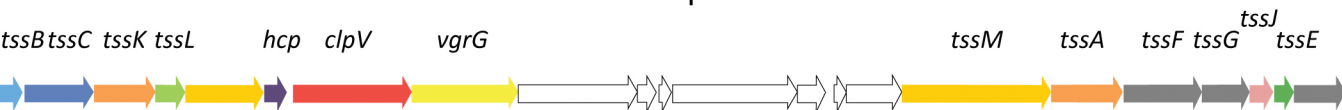

Group 4B

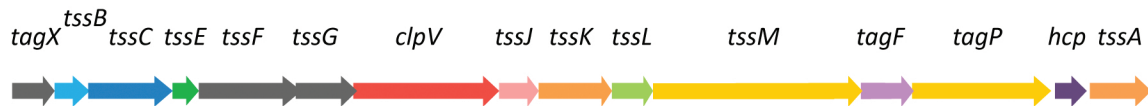

Supplement: Supplementary Figure S1 [file ismej2016169x1.pdf]

Figure S2

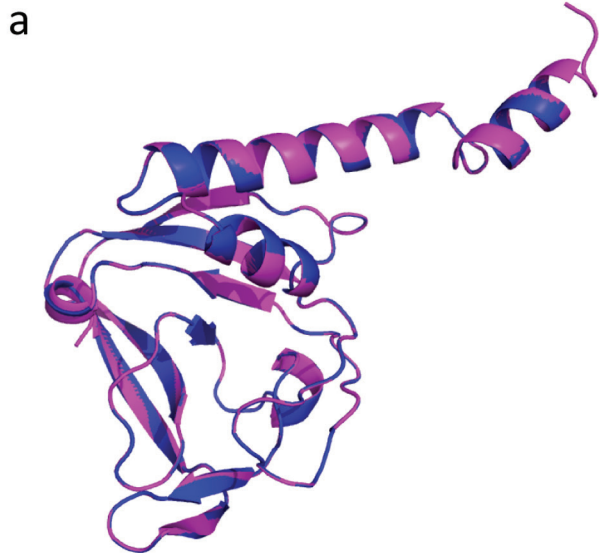

■ Tke1  
■ Tse6

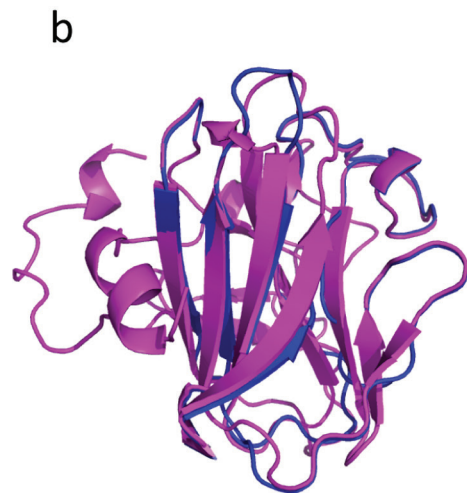

■ Tke3  
■ B30.2 (TRIM20)

Supplement: Supplementary Figure S2 [file ismej2016169x2.pdf]

# Figure S3

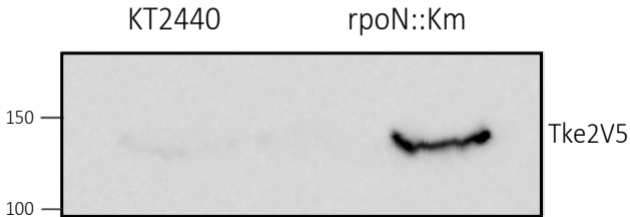

Supplement: Supplementary Figure S3 [file ismej2016169x3.pdf]

## Figure S4

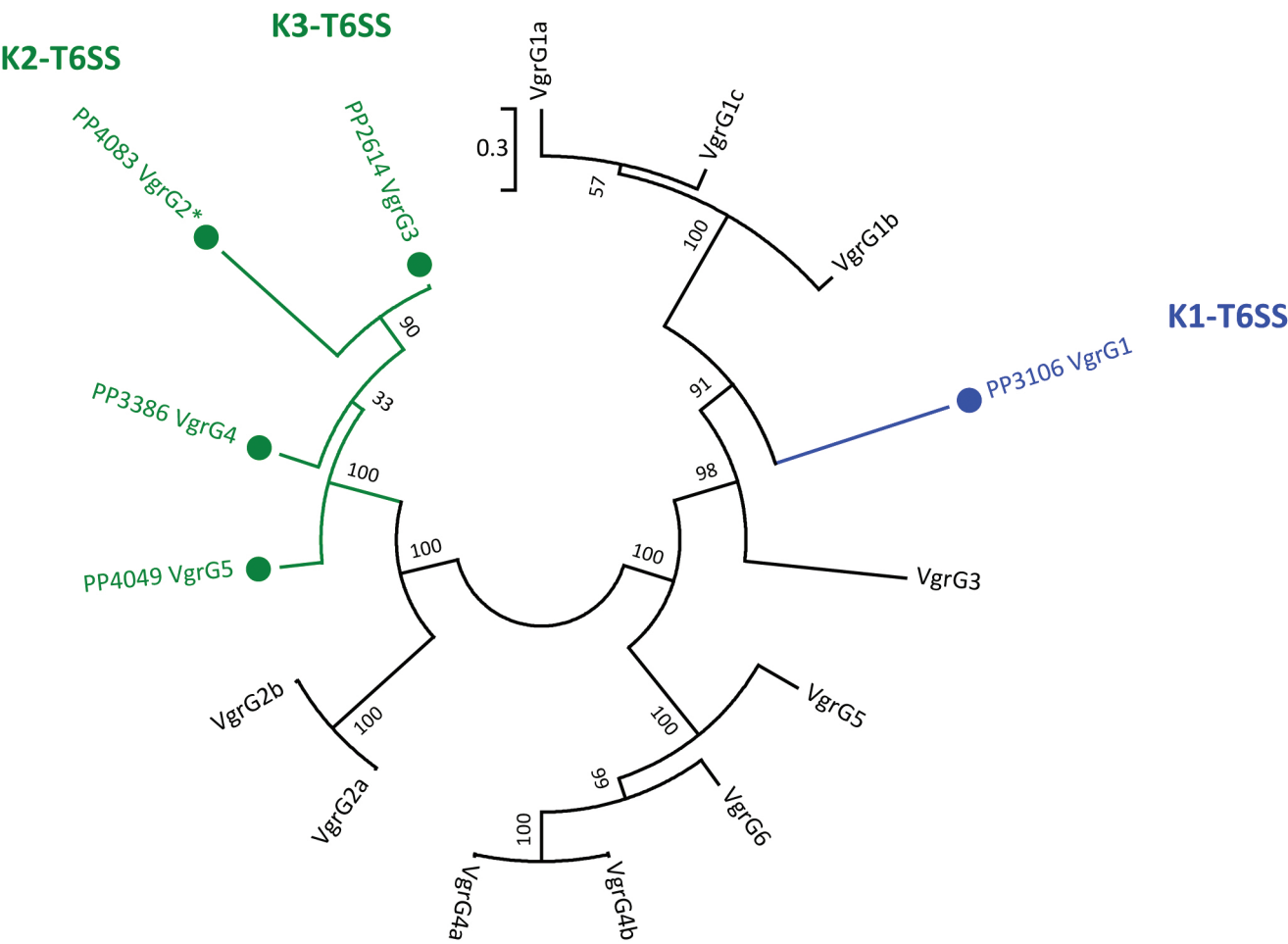

Supplement: Supplementary Figure S4 [file ismej2016169x4.pdf]

Figure S5

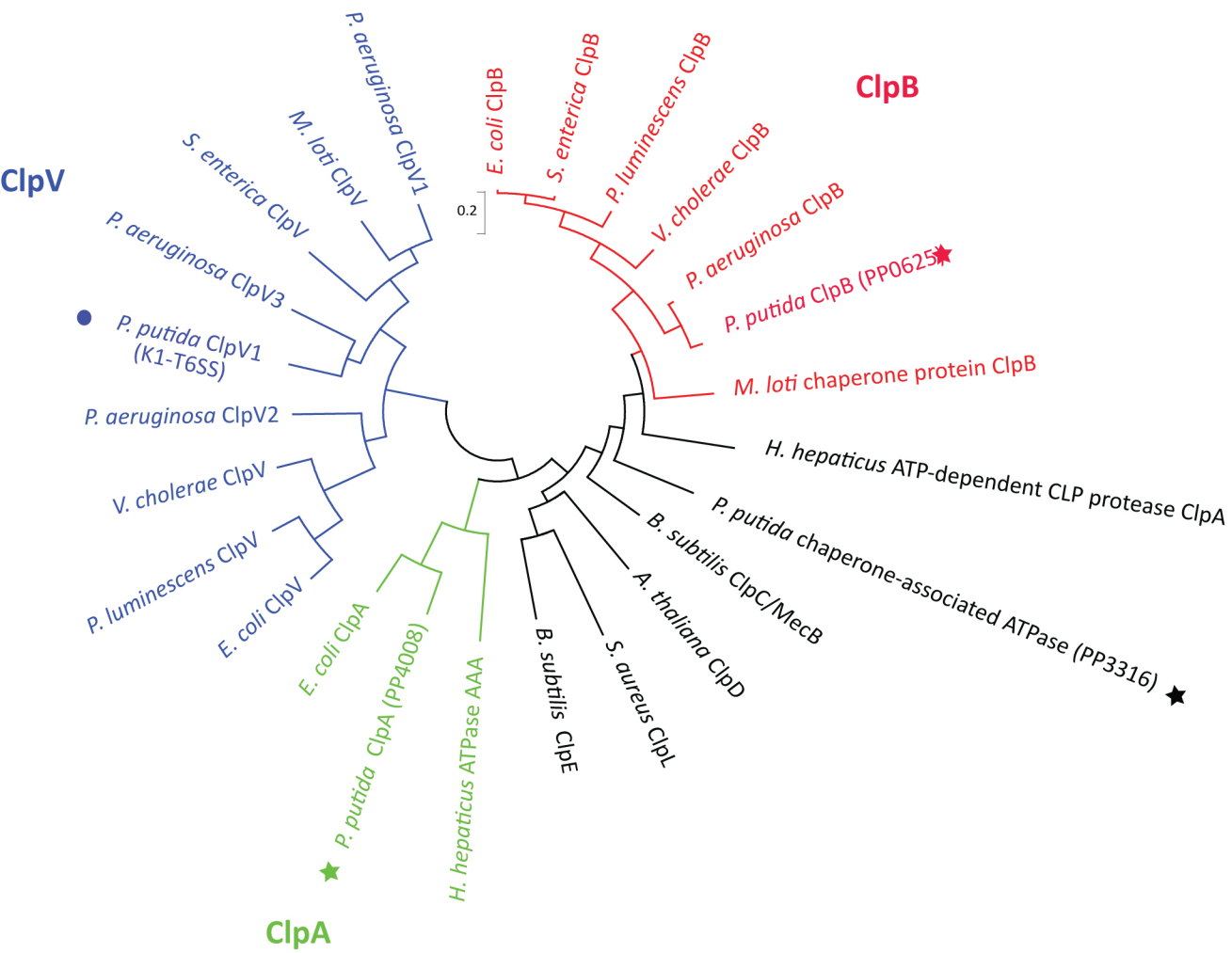

Supplement: Supplementary Figure S5 [file ismej2016169x5.pdf]

# Figure S6

a

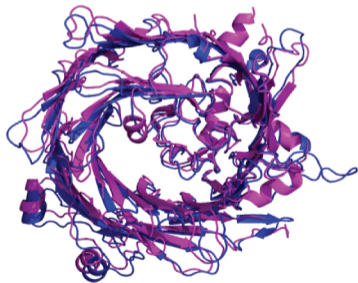

b

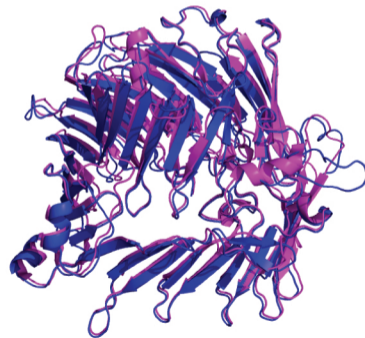

■ Rhs-Tke2  
■ Rhs-ABC toxin

Supplement: Supplementary Figure S6 [file ismej2016169x6.pdf]
