# Supplementary material for: The Pseudomonas putida T6SS is a plant warden against phytopathogens
Source: ISME J. 2017 Jan 3;11(4):972–87. doi: 10.1038/ismej.2016.169 (PMC5363822; doi:10.1038/ismej.2016.169)
Supplement: Supplementary Figure Legends [file ismej2016169x7.docx]

**Supplementary Figures legends**

**Figure S1.** **T6SS clusters in *P. putida.*** Genetic architecture of the three most represented T6SS clusters belonging to phylogenetic groups 1.2, 2 or 4B present in *P. putida* strains. The colour code of the genes correlates with the colour code shown in Figure 1a.

**Figure S2.** **Structural alignment of the *P. putida* KT2440 Tke1 and Tke3 effectors.** Tke1 **(a)** and Tke3 **(b)** structural models are in blue and the structure of the *P. aeruginosa* Tse6 effector (PDB: 4ZV4) **(a)** and the B30.2 fragment of the human protein TRIM20 (PDB: 4CG4) **(b)** in magenta.

**Figure S3. Production of the Tke2 effector.** *P. putida* KT2440 and the isogenic *rpoN*::Km mutant strains bearing a *tke2*-V5 gene were grown in LB medium overnight. Tke2-V5 was detected in whole cell extracts using a monoclonal anti-V5 antibody. The position of the molecular weight markers is indicated on the left.

**Figure S4. Phylogenetic distribution of VgrG proteins.** The distribution of *P. putida* KT2440 (green and blue) and *P. aeruginosa* PAO1 (black) VgrG proteins is shown. The tree was built using maximum-likelihood with 1000 bootstrap replicates using Mega 6. *P. putida* VgrG2 and VgrG3 grouped together and close to orphan VgrG4 and VgrG5 proteins (green). *P. putida* VgrG1 (blue) is not related to the other *P. putida* VgrG proteins and clustered closer to the *P. aeruginosa* VgrG1 protein. The asterisk (*) in the VgrG2 protein indicates that contains a premature stop codon.

**Figure S5. Phylogram of Clp proteins.** The phylogenetic tree shows the distances between different types of Clp proteins including CplV (blue), ClpB (red) and ClpA (green) in different organisms. *P. putida* KT2440 ClpV1 protein is highlighted with a blue circle and it is the sole KT2440 Clp protein belonging to the ClpV group. Other *P. putida* KT2440 Clp proteins are highlighted with a start. Maximum-likelihood tree was built with Mega 6.

**Figure S6. Structural alignment of *P. putida* KT2440 Tke2 Rhs-domain effector.** The structural model of the Rhs domain of the Tke2 effector (blue) is aligned with the structure of the Rhs-repeat of the *Yersinia entomophaga* ABC toxin (magenta, PDB: 4IGL). Panel (a) shows a top view of the structure and panel (b) a side view.
