# Supplementary material for: The Pseudomonas putida T6SS is a plant warden against phytopathogens
Source: ISME J. 2017 Jan 3;11(4):972–87. doi: 10.1038/ismej.2016.169 (PMC5363822; doi:10.1038/ismej.2016.169)
Supplement: Supplementary Table S1 [file ismej2016169x8.docx]

**Table S1:** Bacterial strains and plasmids used in this work

| Bacterial strain | Description^a^ | Source or reference |
| --- | --- | --- |
|  |  |  |
| ***E. coli*** |  |  |
| DH5α | supE44ΔlacU169 (φ80 lacZΔM15) hsdR1 recA1 endA1 gyrA96 thi-1 relA1; Nal^R^ | (Hanahan 1983) |
| Top10 | F−mcrA Δ(mrr-hsdRMS-mcrBC) Φ80lacZΔM15 ΔlacX74 recA1 araD139 Δ(ara leu) 7697 galU galK rpsL (StrR) endA1 nupG | Invitrogen |
| HB101 | supE44 hsdS20 recA13 ara-14 proA2 lacY1 galK2 rpsL20 xyl-5 mtl-1 | (Boyer & Roulland-Dussoix 1969) |
| CC118λ*pir* | Host strain for pKNG101 replication; Δ(ara-leu) araD ΔlacX74 galE galK-phoA20 thi-1 rpsE rpoB argE recA1, lysogenized with λ*pir*; Rif^R^ | (Herrero et al. 1990) |
|  |  |  |
| ***P. putida*** |  |  |
| KT2440R | Wild-type strain ; Rif^R^ | (Espinosa-Urgel et al. 2000) |
| *tssA1*-Tn5 | KT2440R carrying a miniTn5-Km in the *tssA1* (PP3088) gene; Rif^R^, Km^R^ | (Molina-Henares et al. 2010) and this work |
| *tssL1*-Tn5 | KT2440R carrying a miniTn5-Km in the *tssL1* (PP3092) gene; Rif^R^, Km^R^ | (Molina-Henares et al. 2010) and this work |
| *tssK1*-Tn5 | KT2440R carrying a miniTn5-Km in the *tssK1* (PP3093) gene; Rif^R^, Km^R^ | (Molina-Henares et al. 2010) and this work |
| *tssG1*-Tn5 | KT2440R carrying a miniTn5-Km in the *tssG1* (PP3096) gene; Rif^R^, Km^R^ | (Molina-Henares et al. 2010) and this work |
| *tssF1*-Tn5 | KT2440R carrying a miniTn5-Km in the *tssF1* (PP3097) gene; Rif^R^, Km^R^ | (Molina-Henares et al. 2010) and this work |
| *tssE1*-Tn5 | KT2440R carrying a miniTn5-Km in the *tssE1* (PP3098) gene; Rif^R^, Km^R^ | (Molina-Henares et al. 2010) and this work |
| Δ*tssA1* | Markerless KT2440R null mutant in the *tssA1* (PP3088) gene, disabling the K1-T6SS; Rif^R^ | This work |
| Δ*tssA1*Δ*tssM2*Δ*tssM3* (ΔT6SS) | Markerless KT2440R null mutant in the *tssA1/tssM2/tssM3* (PP3088/PP4071/PP2627) genes disabling all three T6SS clusters; Rif^R^ | This work |
| miniCTX-*hcp1*-HA | KT2440R containing a miniCTX transposon expressing a HA tagged *hcp1* gene; Rif^R^, Tc^R^ | This work |
| Δ*tssA1* miniCTX-*hcp1*-HA | *tssA1* null mutant containing a miniCTX transposon expressing a HA tagged *hcp1* gene; Rif^R^, Tc^R^ | This work |
| *rpoN*::Km | KT2440R carrying a Km cassette in the *rpoN* (PP0952) gene; Km^R^ | (Köhler et al. 1989) |
| *rpoN*::Km *tke2*-V5 | *rpoN*::Km mutant in which the *tke2* gene has been replaced by a version tagged with a dual V5 epitope; Km^R^ | This work |
| *rpoN*::Km Δ*tssA1* *tke2*-V5 | *rpoN*::Km *tke2*-V5 containing the Δ*tssA1* mutation; Km^R^ | This work |
|  |  |  |
| **Other strains** |  |  |
| *Xanthomonas campestris pv. campestris* IVIA 2734-1 | Wild-type strain | María Milagros Lopez collection (IVIA, Spain) |
| *Agrobacterium tumefaciens* C58 | Wild-type strain | Erh Min Lai collection (Academia Sinica, Taiwan) |
| *Pectobacterium carotovorum subsp. carotovorum* SCRI 194 | Wild-type strain | María Milagros Lopez collection (IVIA, Spain) |
| *Pseudomonas syringae pv tomato* DC3000 | Wild-type strain | Martin Buck collection (Imperial College London, UK) |
|  |  |  |
|  |  |  |
|  |  |  |
|  |  |  |
|  |  |  |
| **Plasmids** |  |  |
| pCR-BluntII-TOPO | Blunt cloning vector; Ap^R^, Km^R^ | Invitrogen |
| pRK600 | Helper plasmid; oriColE1 mobRK2 traRK2; Cm^R^ | (Kessler et al. 1992) |
| pRK2013 | Helper plasmid; oriColE1 mobRK2 traRK2; Km^R^ | (Figurski & Helinski 1979) |
| pKNG101 | Gene replacement suicide vector, *ori*R6K, *oriT*RK2, *sacB;* Sm^R^ | (Kaniga et al. 1991) |
| pBAD33 | Cloning vector containing the pBAD promoter inducible by L-arabinose; p15A origin; Cm^R^ | (Guzman et al. 1995) |
| pNDM220 | Low copy number cloning vector containing the lacI^q^ gene and the LacI^q^-regulated promoter PA1/O4/O3, IPTG-inducible; Ap^R^ | (Gotfredsen & Gerdes 1998) |
| miniCTX-1 | Plasmid for the integration of genes into the att site of the P. putida chromosome; Tc^R^ | (Hoang et al. 2000) |
| pRL662-gfp2 | Broad host range vector derived from pBBR1MCS-2 expressing a green fluorescence protein (gfp); Gm^R^ | Erh-Min Lai collection |
| pTki2 | pBAD33 carrying in SacI-XbaI a 550 bps PCR fragment containing the entire *P. putida* *tki2* (PP3108.1) gene; Cm^R^ | This work |
| pTke2-CT | pNDM220 carrying in BamHI-XhoI a 514 bps PCR fragment containing the C-terminal part of the *P. putida* *tke2* (PP3108) gene; Ap^R^ | This work |
| pK*tssA1* | pKNG101 carrying in XbaI-BamHI a 1.7-Kb PCR fragment containing the regions up- and downstream the *P. putida tssA1* (PP3088) gene; Sm^R^ | This work |
| pK*tssM2* | pKNG101 carrying in XbaI-BamHI a 1.6-Kb PCR fragment containing the regions up- and downstream the *P. putida tssM2* (PP4071) gene; Sm^R^ | This work |
| pK*tssM3* | pKNG101 carrying in XbaI-BamHI a 1.6-Kb PCR fragment containing the regions up- and downstream the *P. putida tssM3* (PP2627) gene; Sm^R^ | This work |
| pK*tke2V5* | pKNG101 carrying in XbaI-BamHI a 1.2-Kb PCR fragment containing a C-terminally V5 dual-tagged *P. putida* *tke2* gene and the regions up- and downstream this gene; Sm^R^ | This work |
| miniCTX-Plac-hcp1-HA | miniCTX-1 carrying in EcoRI-BamHI a 0.6-Kb PCR fragment encoding a C-terminal HA-tagged *hcp1* gene from a Plac promoter ; Tc^R^ | This work |

^a^ Ap^R^, Gm^R^, Km^R^, Nal^R^, Rif^R^, Sm^R^ and Tc^R^, resistance to ampicillin, gentamycin, kanamycin, nalidixic acid, rifampicin, streptomycin and tetracycline, respectively

**References**

Boyer HW, Roulland-Dussoix D. (1969). A complementation analysis of the restriction and modification of DNA in Escherichia coli. J. Mol. Biol. 41:459–72.

Espinosa-Urgel M, Salido A, Ramos JL. (2000). Genetic analysis of functions involved in adhesion of Pseudomonas putida to seeds. J. Bacteriol. 182:2363–9.

Figurski DH, Helinski DR. (1979). Replication of an origin-containing derivative of plasmid RK2 dependent on a plasmid function provided in trans. Proc. Natl. Acad. Sci. U.S.A. 76:1648–52.

Gotfredsen M, Gerdes K. (1998). The Escherichia coli relBE genes belong to a new toxin-antitoxin gene family. Mol. Microbiol. 29:1065–76.

Guzman LM, Belin D, Carson MJ, Beckwith J. (1995). Tight regulation, modulation, and high-level expression by vectors containing the arabinose PBAD promoter. J. Bacteriol. 177:4121–30.

Hanahan D. (1983). Studies on transformation of Escherichia coli with plasmids. Journal of molecular biology 166:557–80.

Herrero M, de Lorenzo V, Timmis KN. (1990). Transposon vectors containing non-antibiotic resistance selection markers for cloning and stable chromosomal insertion of foreign genes in gram-negative bacteria. J. Bacteriol. 172:6557–67.

Hoang TT, Kutchma AJ, Becher A, Schweizer HP. (2000). Integration-proficient plasmids for Pseudomonas aeruginosa: site-specific integration and use for engineering of reporter and expression strains. Plasmid 43:59–72.

Kaniga K, Delor I, Cornelis GR. (1991). A wide-host-range suicide vector for improving reverse genetics in gram-negative bacteria: inactivation of the blaA gene of Yersinia enterocolitica. Gene 109:137–41.

Kessler B, de Lorenzo V, Timmis KN. (1992). A general system to integrate lacZ fusions into the chromosomes of gram-negative eubacteria: regulation of the Pm promoter of the TOL plasmid studied with all controlling elements in monocopy. Mol. Gen. Genet. 233:293–301.

Köhler T, Harayama S, Ramos JL, Timmis KN. (1989). Involvement of Pseudomonas putida RpoN sigma factor in regulation of various metabolic functions. J. Bacteriol. 171:4326–33.

Molina-Henares MA, de la Torre J, García-Salamanca A, Molina-Henares AJ, Herrera MC, Ramos JL, et al. (2010). Identification of conditionally essential genes for growth of Pseudomonas putida KT2440 on minimal medium through the screening of a genome-wide mutant library. Environ. Microbiol. 12:1468–85.
