# Supplementary material for: The Pseudomonas putida T6SS is a plant warden against phytopathogens
Source: ISME J. 2017 Jan 3;11(4):972–87. doi: 10.1038/ismej.2016.169 (PMC5363822; doi:10.1038/ismej.2016.169)
Supplement: Supplementary Table S2 [file ismej2016169x9.docx]

| **Table S2:** Sequence of the primers used in this work | | | |  |
| --- | --- | --- | --- | --- |
| Amplified/Deleted gene and promoter region from  *P. putida* KT2440 | Plasmid | Name | Sequence (5’ 🡪 3’)^a^ | |
|  |  |  |  | |
| PP3088 (*tssA1*) | pK*tssA1* | tssA1-1 | GGAA**TCTAGA**CAACAACCGCGAACGCAG | |
|  |  | tssA1-2 | CGGCC**GAATTC**CATAGAACACTCTGGACAT | |
|  |  | tssA1-3 | GCCGG**GAATTC**TAGGGGCTGCAATAAAAG | |
|  |  | tssA1-4 | TCGGTGAACA**GGATCC**AGTC | |
| PP4071 (*tssM2*) | pK*tssM2* | tssM2-1 | TAGAG**TCTAGA**GACGCGCCGAGCCATC | |
|  |  | tssM2-2 | CTACATGACTTGATTCATCGAGGCTCC | |
|  |  | tssM2-3 | ATGAATCAAGTCATGTAGGCAGGAGGC | |
|  |  | tssM2-4 | TTCAA**GGATCC**GCGTGAACGCTCGTTACA | |
| PP2627 (*tssM3*) | pK*tssM3* | tssM3-1 | AGGAA**TCTAGA**ACGACGCTACCGGCTACC | |
|  |  | tssM3-2 | TCATAGTCGTTGATTCATCGAGGCTCC | |
|  |  | tssM3-3 | ATGAATCAACGACTATGAACCTCGTCA | |
|  |  | tssM3-4 | ACCTT**GGATCC**TGAGCTGACGCTGCACAT | |
| PP3108 (*tke2*) | pK*tke2V5* | tke2V5-1 | GGTCC**TCTAGA**AGAATCTGCGCTTCCAAGGT | |
|  |  | tke2V5-2 | aggcttacccgtagaatcgagaccgaggagagggttagggataggcttaccCCATACATCAACTCCTTTAATTACT | |
|  |  | tke2V5-3 | gattctacgggtaagcctatccctaaccctctcctcggtctcgattctacgTGAATTAAAGGAGTTGATGTATGG | |
|  |  | tke2V5-4 | AATAA**GGATCC**CGGACACCTGCAAAATAC | |
| PP3108 (*tke2*) | pTke2-CT | Tke2-F | CCGGC**GGATCC**taacaggaggaattaaccATGCGTTATGTCACTCAGGACC | |
|  |  | Tke2-R | CCGGG**CTCGAG**CTAcgtagaatcgagaccgaggagagggttagggataggcttaccCCATACATCAACTCCTTTAATTAC | |
| PP3108.1 (*tki2*) | pTki2 | Tki2-F | CCGCC**GAGCTC**taacaggaggaattaaccATGGTAATCAATGGCGGTTCATTGG | |
|  |  | Tki2-R | AATTA**TCTAGA**ttagcacgcgtagtccggcacgtcgtacgggtaAGCCCCAAGACCTGTCAACTTGAT | |
| PP3089 (*hcp1-*HA)^b^ | miniCTX-Plac-hcp1-HA | Hcp1HA-F | CCGCC**GAATTC**taacaggaggaattaaccATGTTGTTAATGGAGAGTTT | |
|  |  | Hcp1HA-R | AACTT**GGATCC**TTAGCACGCGTAGTCCGGCACGTC | |

^a^ The sequences of the restriction sites are indicated in bold and the annealing region is underlined. Artificial Shine-Dalgarno and V5-tag are shown in lowercase.

^b^ The strain used as a template for this PCR reaction contains the gene encoding Hcp1 with a C-terminal HA-tag.
