# Supplementary material for: The Pseudomonas putida T6SS is a plant warden against phytopathogens
Source: ISME J. 2017 Jan 3;11(4):972–87. doi: 10.1038/ismej.2016.169 (PMC5363822; doi:10.1038/ismej.2016.169)
Supplement: Supplementary Table S3 [file ismej2016169x10.docx]

**Table S3:** Distribution of T6SS loci in *Pseudomonas putida strains*

| ***P. putida* strain** | **T6SS clusters** | **Group 1.1** | **Group 1.2** | **Group 2** | **Group 3** | **Group 4A** | **Group 4B** | **Group 5** | **VgrGs** | **Hcps** |
| --- | --- | --- | --- | --- | --- | --- | --- | --- | --- | --- |
| **PA14H7** | 4 |  | 1 | 2 | 1 |  |  |  | 7 | 3 |
| **TRO1** | 3 |  | 1,Partial | 1 |  |  | 1 |  | 4 | 2 |
| **KT2440** | 3 |  | 2  (K2, K3) |  |  |  | 1  (K1) |  | 5 | 6 |
| **DOT-T1E** | 3 |  | 2 |  |  |  | 1 |  | 5 | 2 |
| **H** | 3 |  | 2 |  |  |  | 1 |  | 4 | 5 |
| **LS46** | 3 |  | 2 |  |  |  | 1 |  | 5 | 3 |
| **B6-2** | 3 |  | 2 |  |  |  | 1 |  | 4 | 3 |
| **GB-1** | 2 |  | 1 |  |  |  | 1 |  | 4 | 5 |
| **F1** | 2 |  | 1 |  |  |  | 1 |  | 3 | 2 |
| **ND6** | 2 |  | 1 |  |  |  | 1 |  | 3 | 3 |
| **S13.1.2** | 2 |  | 1 |  |  |  | 1 |  | 4 | 2 |
| **SJTE-1** | 2 |  | 1 |  |  |  | 1 |  | 2 | 2 |
| **str. Idaho** | 2 |  | 1 |  |  |  | 1 |  | 5 | 3 |
| **YKD221** | 2 |  | 1 |  |  |  | 1 |  | 3 | 2 |
| **791_PPUT** | 2 |  | 1 |  |  |  | 1 |  | 5 | 2 |
| **T2-2** | 2 |  | 1 |  |  |  | 1 |  | 3 | 5 |
| **W619** | 2 |  |  | 1 |  |  | 1 |  | 3 | 4 |
| **CBB5** | 2 |  |  | 1 |  |  | 1 |  | 5 | 2 |
| **SQ1** | 2 |  |  | 1 |  |  | 1 |  | 4 | 4 |
| **CSV86** | 2 |  | 1 |  |  | 1 |  |  | 9 | 3 |
| **W15Oct28** | 2 |  | 1 | 1 |  |  |  |  | 6 | 2 |
| **S16** | 1 |  |  |  |  |  | 1 |  | 3 | 3 |
| **NBRC 14164** | 1 |  |  |  |  |  | 1 |  | 3 | 2 |
| **HB3267** | 1 |  | Partial |  |  |  | 1 |  | 3 | 6 |
| **DLL-E4** | 1 |  |  |  |  |  | 1 |  | 3 | 3 |
| **PD1** | 1 |  |  |  |  |  | 1 |  | 1 | 3 |
| **SF1** | 1 |  | Partial |  |  |  | 1 |  | 5 | 3 |
| **LF54** | 1 |  |  |  |  |  | 1 |  | 3 | 2 |
| **ATH-43** | 1 |  |  |  |  |  | 1 |  | 1 | 2 |
| **UASWS0946** | 1 | 1 |  |  |  |  |  |  | 3 | 1 |
| **S12** | 1 |  | 1 |  |  |  |  |  | 1 | 1 |
| **B001** | 1 |  | 1 |  |  |  |  |  | 2 | 1 |
| **SJ3** | 1 |  | 1 |  |  |  |  |  | 4 | 1 |
| **S610** | 1 |  | 1 |  |  |  |  |  | 2 | 1 |
| **MC4-5222** | 1 |  |  | 1 |  |  |  |  | 7 | 1 |
| **KG-4** | 1 |  |  |  | 1 |  |  |  | 2 | 1 |
| **MTCC 5279** | 1 |  |  |  |  |  |  | 1 | 2 | 0 |
| **BIRD-1** | 0 |  | Partial |  |  |  |  |  | 0 | 0 |
| **H8234** | 0 |  |  |  |  |  |  |  | 2 | 0 |
