# Supplementary material for: The Pseudomonas putida T6SS is a plant warden against phytopathogens
Source: ISME J. 2017 Jan 3;11(4):972–87. doi: 10.1038/ismej.2016.169 (PMC5363822; doi:10.1038/ismej.2016.169)
Supplement: Supplementary Table S4 [file ismej2016169x11.docx]

**Table S4:** Characteristics of proteins encoded by the *P. putida* KT2440 K1-T6SS cluster.

| Locus name | Protein name | Identities with T6SS PAO1/Other identities | Conserved Domains  COG/pfam/TIGR  (Short Name)/Phyre (P)^a^ | Molecular weight  (KDa)/pI^b^ | Predicted cellular location^c, d^ | Transmembrane Helices^e^ | Predicted signal peptide^f, g^ |
| --- | --- | --- | --- | --- | --- | --- | --- |
| **Structural operon** | | | | | | | |
| PP3088 | TssA1 | PA0082 (HsiA1)  72/316 (23%) | COG3515/pfam06812 (ImpA-rel_N) + TIGR03363 (VI_chp_8) | 361 a.a.  39.9/4.4 | 1. Unknown  2. Cytoplasmic | 0 | No  Yes (1-18) |
| PP3089 | TssD1/Hcp1 | PA0085 (Hcp1)  53/169 (31%) | COG3157 (Hcp)/ pfam05638 (DUF796)/ TIGR03344 (VI_effect_Hcp1) | 180 a.a.  19.5/5.3 | 1. Extracellular  2. Cytoplasmic | 0 | No  No |
| PP3090 | TagP1 | PA0077 (IcmF1)  151/406 (37%) | COG3523 (IcmF)/ pfam14331 (ImcF-related_N)/TIGR03348 (VI_IcmF) + pfam00691 (OmpA) | 831 a.a.  93.1/9 | 1. Inner Membrane  2. Inner Membrane | 3 | No  No |
| **PP3090.1** | TagF1 | PA0076 (TagF1)  8/22  (36%) | pfam (DUF2094) | 302 a.a.  33.8/6.2 | 1. Cytoplasmic  2. Unknown | 0 | No  No |
| PP3091 | TssM1 | PA0077 (IcmF1)  152/466 (33%) | COG3523 (IcmF)/ pfam14331 (ImcF-related_N)/TIGR03348 (VI_IcmF) | 1267 a.a.  138.7/6.8 | 1. Inner Membrane  2. Inner Membrane | 3 | No  No |
| PP3092 | TssL1 | PA0078 (TssL1)  50/187 (27%) | COG3455/pfam09850 (DUF2077)/ TIGR03349 (TIGR03349) | 238 a.a.  26.9/5.7 | 1. Cytoplasmic  2. Inner Membrane | 1 | No  No |
| PP3093 | TssK1 | PA0079 (TssK1)  140/450 (31%) | COG3522/ pfam05936 (DUF876)/TIGR03353 (VI_chp_4) | 447 a.a.  50/6.4 | 1. Cytoplasmic  2. Unknown | 0 | No  No |
| PP3094 | TssJ1 | PA0080 (TssJ1)  33/107 (31%) | COG3521/pfam12790 (T6SS-SciN)/TIGR03352 (VI_chp_3) | 240 a.a.  25.9/4.7 | 1. Unknown  2. Periplasm | 0 | No  Yes (1-26) |
| PP3095 | TssH1/  ClpV1 | PA0090 (ClpV1)  452/893 (51%) | COG0542 (ClpA) + pfam07724 (AAA_2)/ TIGR03345 (VI_ClpV1) | 878 a.a.  96.5/6.1 | 1. Cytoplasmic  2. Unknown | 0 | No  Yes (1-22) |
| PP3096 | TssG1 | PA0089 (TssG1)  111/328 (34%) | COG3520/pfam06996 (DUF1305)/TIGR03347 (VI_chp_1) | 356 a.a.  40.8/9.9 | 1. Cytoplasmic  2. Cytoplasmic | 0 | No  No |
| PP3097 | TssF1 | PA0088 (TssF1)  216/632 (34%) | COG3519/pfam05947 (DUF879)/TIGR03359 (VI_chp_6) | 606 a.a.  69.2/7.3 | 1. Cytoplasmic  2. Cytoplasmic | 0 | No  No |
| PP3098 | TssE1 | PA0087 (TssE1)  42/138 (30%) | COG3518/ pfam04965 (GPW_gp25)/ TIGR03357 (VI_zyme) | 160 a.a.  18.4/8.5 | 1. Cytoplasmic  2. Cytoplasmic | 0 | No  No |
| PP3099 | TssC1 | PA0084 (TssC1)  251/486 (52%) | COG3517/pfam05943 (DUF877)/TIGR03355 (VI_chp_2) | 500 a.a.  56.1/4.9 | 1. Cytoplasmic  2. Cytoplasmic | 0 | No  No |
| PP3100 | TssB1 | PA0083 (TssB1)  68/161 (42%) | COG3516/pfam05591 (DUF770)/TIGR03358 (VI_chp_5) | 191 a.a.  21.7/7.7 | 1. Cytoplasmic  2. Cytoplasmic | 0 | No  No |
| **PP3100.1** | TagX1 |  | -- | 254 a.a.  28.5/5.5 | 1. Cytoplasmic  2. Unknown | 0 | No  No |
| **Intermediate region** | | | | | | | |
| **PP3100.2** |  |  | -- | 68 a.a.  7.15/9.26 | 1. Unknown  2. Periplasm | 0 | No  No |
| **PP3100.3** |  |  | -- | 183 a.a.  20.6/9.04 | 1. Inner Membrane  2. Inner Membrane | 3 | No  Yes (1-40) |
| PP3101 |  |  | COG1397 (DraG)/ pfam03747 (ADP_ribosyl_G)/ TIGR02662 (dinitro_DRAG) | 260 a.a.  28.7/6.5 | 1. Unknown  2. Unknown | 0 | No  No |
| **PP3101.1** |  |  | -- | 90 a.a.  10.2/4.81 | 1. Unknown  2. Cytoplasmic | 0 | No  No |
| **PP3101.2** |  |  | -- | 189 a.a.  21.5/10.7 | 1. Unknown  2. Cytoplasmic | 0 | No  No |
| **PP3101.3** | Partial | PP3106  28/61 (46%) | -- | 112 a.a.  12.8/9.5 | 1. Unknown  2. Unknown | 0 | No  No |
| **PP3101.4** | Partial | PP3105  100/231 (43%) | -- | 232 a.a.  26.2/9.94 | 1. Inner membrane  2. Inner membrane | 6 | No  No |
| **PP3101.5** | Partial | PP3106 31/51 (61%) | Pfam05954 (Phage GPD) | 67 a.a.  7.6/9.30 | 1. Unknown  2. Cytoplasmic | 0 | No  No |
| **PP3101.6** | Partial |  | COG0013: Alanyl-tRNA synthetase | 103 a.a.  11.9/5.9 | 1. Unknown  2. Cytoplasmic | 0 | No  No |
| **PP3101.7** |  |  | -- | 101 a.a.  11.1/6.31 | 1. Unknown  2. Cytoplasmic | 0 | No  No |
| **PP3101.8** | Partial |  | COG3344/  pfam00078/  TIGR04416/  (RVT_1)  Group II intron reverse transcriptase | 129 a.a.  14.8/10.5 | 1. Unknown  2. Cytoplasmic | 0 | No  No |
| **PP3101.9** | Partial |  | Ribosomal protein S7 cl00313:uS7 Superfamily | 58 a.a.  6.7/10.15 | 1. Unknown  2. Unknown | 0 | No  No |
| PP3102 | Tki1 | PA0092 (Tsi6) 44/93  (47%) | P:Tsi6  (4-89 a.a.) C:100% | 98 a.a.  10.9/8.4 | 1. Unknown  2. Cytoplasmic | 0 | No  No |
| PP3103 | Tke1 | PA0093 (Tse6) 88/160 (55%) | pfam (Toxin_61)  P: Tse6  (227-383 a.a.) C: 100% | 391 a.a.  42.6/9.9 | 1. Unknown  2. Inner Membrane | 0 | No  No |
| **PP3103.1** |  |  | -- | 98 a.a.  11.5/9.1 | 1. Unknown  2. Inner Membrane | 1 | No  No |
| **VgrG1 operon** | | | | | | | |
| PP3104 |  |  | -- | 338 a.a.  35.1/9.5 | 1. Unknown  2. Inner Membrane | 1 | No  No |
| PP3105 |  | PP3101.4  100/231 (43%) | -- | 231 a.a.  26.5/10.5 | 1. Inner Membrane  2. Inner Membrane | 4 | No  No |
| PP3106 | TssI1/VgrG1 | PA0091 (VgrG1)  213/588 (36%) | COG3501 (VgrG)/ pfam05954 (Phage_GPD)/ TIGR03361 (VI_Rhs_Vgr) | 618 a.a.  69.5/5.6 | 1. Cytoplasmic  2. Extracellular | 0 | No  No |
| **PP3106.1** | EagR1a | PA0094  6/14  (43%) | pfam08786 (DUF1795) | 194 a.a.  21.6/5.8 | 1. Cytoplasmic  2. Unknown | 0 | No  No |
| PP3107 | EagR1b | PA0094  39/141 (28%) | COG5435/pfam08786 (DUF1795) | 173 a.a.  19.1/4.9 | 1. Unknown  2. Extracellular | 0 | No  No |
| PP3108 | Tke2 |  | pfam05488 (PAAR_motif) + COG3209 (RhsA)/ TIGR03696 (Rhs_assc_core) + HNH nuclease (SM00507) / P: Endonuclease (1331-1362 a.a.) C: 50% | 1385 a.a.  155.8/6 | 1. Unknown  2. Inner Membrane | 2 | No  No |
| **PP3108.1** | Tki2 |  | -- | 158 a.a.  17.4/4.6 | 1. Cytoplasmic  2. Cytoplasmic | 0 | No  No |
| **PP3108.2** | Partial | PP3108  207/219 (95%) | pfam05593 (RHS-repeat) | 278 a.a.  31.2/5.4 | 1. Unknown  2. Extracellular | 0 | No  No |
| **PP3108.3** |  |  | pfam14136 (DUF4303) | 176 a.a.  20.4/4.5 | 1. Unknown  2. Cytoplasmic | 0 | No  No |
| PP3109 | Partial | PP3108  24/26 (92%) | pfam03527 (RHS) | 143 a.a.  16.1/11.1 | 1. Inner Membrane  2. Inner Membrane | 1 | No  No |
| **PP3109.1** |  |  | P: Multiheme cytochromes (28-120 a.a.) C: 65% | 159 a.a.  18.2/5.1 | 1. Cytoplasmic  2. Cytoplasmic | 0 | No  No |
| **PP3109.2** | Partial |  | pfam14427 (Pput2613-deam) | 76 a.a.  8.1/5.8 | 1. Unknown  2. Cytoplasmic | 0 | No  No |
| **PP3109.3** | Partial |  | Pfam15588 (Imm7) | 75 a.a.  8/4.1 | 1. Unknown  2. Cytoplasmic | 0 | No  No |
| **PP3109.4** | Tke3 |  | P: B30.2 domain of TRIM20 (103-198 a.a.)  C: 84% | 199 a.a.  22.3/5.2 | 1. Cytoplasmic  2. Unknown | 0 | No  No |
| **PP3109.5** | Tki3 |  | Pfam15428 (Imm14) | 173 a.a.  20/8.3 | 1. Cytoplasmic  2. Cytoplasmic | 0 | No  No |
| **PP3109.7** |  |  | -- | 201 a.a.  23.2/6.3 | 1. Cytoplasmic  2. Outer membrane | 0 | No  No |
| PP3110 |  |  | -- | 70 a.a.  8/10 | 1. Unknown  2. Periplasm | 0 | No  No |
| PP3111 |  |  | Pfam09339 (HTH-IclR) | 34 a.a.  3.7/7.6 | 1. Unknown  2. Unknown | 0 | No  No |

a.a.: amino acids

Newly annotated proteins are in bold

Partial proteins or those with premature stop codon are underline

a: Structural-based homology prediction using the Protein Homology/analogy Recognition Engine (Phyre) server (Kelley, et al., 2009). C stands for Confidence.

b: The molecular weight and isoelectric point (pI) are based on prediction by the software ExPASy (http://www.expasy.ch/tools/pi_tool.html).

c: The cellular localization is based on prediction by PSORTb

(http://www.psort.org/psortb/index.html).

d: The cellular localization is based on prediction by SOSUIGramN

(http://bp.nuap.nagoya-u.ac.jp/sosui/sosuigramn/sosuigramn_submit.html).

e: The prediction of transmembrane domains was determined by TMHMN (http://www.cbs.dtu.dk/services/TMHMM/)

f: The prediction of signal peptides was determined by SignalP

(http://www.cbs.dtu.dk/services/SignalP/).

g: The prediction of signal peptides was determined by SOSUIsignal (http://bp.nuap.nagoya-u.ac.jp/sosui/sosuisignal/sosuisignal_submit.html).
