# Supplementary material for: The Pseudomonas putida T6SS is a plant warden against phytopathogens
Source: ISME J. 2017 Jan 3;11(4):972–87. doi: 10.1038/ismej.2016.169 (PMC5363822; doi:10.1038/ismej.2016.169)
Supplement: Supplementary Table S5 [file ismej2016169x12.docx]

**Table S5:** Characteristics of proteins encoded by the *P. putida* KT2440 K2- and K3-T6SS clusters

| Locus name | Protein name | Protein length | Identities with H2-T6SS PAO1 | K2-K3 Identities /Other identities | Conserved Domains  COG/pfam/TIGR  (Short Name)/ Phyre^a^ | Predicted cellular location^b, c^ | Transmem-brane helices^d^ | Predicted signal peptide^e, f^ |
| --- | --- | --- | --- | --- | --- | --- | --- | --- |
| PP4071 | TssM2 | 1208 a.a. | PA1669 (IcmF2) 26% | 82% | COG3523 (IcmF)  pfam06761(IcmF)  TIGR03348 (VI_IcmF) | 1. Inner Membrane  2. Inner Membrane | 3 | No  No |
| PP2627 | TssM3 | 1206 a.a. | 27% |  |  |  |  |  |
| PP4072 | TssA2 | 488 a.a. | PA1656 (HsiA2) 26% | 77% | pfam06812 (ImpA-rel_N)+ TIGR03362 (VI_chp_7) | 1. Cytoplasmic  2. Cytoplasmic | 0 | No  No |
| PP2626 | TssA3 | 487 a.a. | 24% |  |  | 1. Cytoplasmic  2. Inner Membrane |  |  |
| PP4073 | VasI2 | 210 a.a. |  | 64% | Pfam11319 (DUF3121)/ TIGR03360 (VI_minor_1) | 1. Cytoplasmic  2. Unknown | 0 | No  Yes (1-23) |
| PP2625 | VasI3 | 231 a.a. |  |  |  | 1. Cytoplasmic  2. Cytoplasmic | 1 | No  No |
| PP4074 | TssB2 | 167 a.a. | PA1657 (HsiB2) 50% | 82% | COG3516/pfam05591(DUF770)/TIGR03358 (VI_chp_5) | 1. Cytoplasmic  2. Cytoplasmic | 0 | No  No |
| PP2624 | TssB3 | 167 a.a. | 50% |  |  |  |  |  |
| PP4075 | TssC2 (partial) | 428/495*a.a. | PA1658 (HsiC2) 65% | 84% | COG3517/ pfam05943 (DUF877)/ TIGR03355 (VI_chp_2) | 1. Cytoplasmic  2. Cytoplasmic | 0 | No  No |
| PP2623 | TssC3 | 496 a.a. | 67% |  |  |  |  |  |
| PP4076 | TssE2 | 136 a.a. | PA1659  (HsiF) 27% | 88% | COG3518/ pfam04965 (GPW_gp25)/ TIGR03357 (VI_zyme) | 1. Cytoplasmic  2. Unknown | 0 | No  No |
| PP2622 | TssE3 | 136 a.a. | 30% |  |  |  |  |  |
| PP4077 | TssF2 | 588 a.a. | PA1660  (HsiG) 35% | 86% | COG3519/ pfam05947 (DUF879)/ TIGR03359 (VI_chp_6) | 1. Cytoplasmic  2. Cytoplasmic | 0 | No  No |
| PP2621 | TssF3 | 588 a.a. | 34% |  |  |  |  |  |
| PP4078 | TssG2 | 338 a.a. | PA1661  (HsiH) 34% | 88% | COG3520/ pfam06996 (DUF1305)/ TIGR03347 (VI_chp_1) | 1. Cytoplasmic  2. Cytoplasmic | 0 | No  No |
| PP2620 | TssG3 | 338 a.a. | 35% |  |  | 1. Unknown  2. Cytoplasmic |  |  |
| PP2619 | Fha3 | 189 a.a. | PA1665  (Fha2) 25% |  | COG3456/TIGR03354 (VI_FHA) | 1. Unknown  2. Cytoplasmic | 0 | No  No |
| PP4079 | TssJ2 | 265 a.a. | PA1666  (Lip2) 22% | 76% | COG3521/pfam12790 (T6SS-SciN)/TIGR03352 (VI_chp_3) | 1. Unknown  2. Periplasm | 0 | No  Yes (1-27) |
| PP2618 | TssJ3 | 265 a.a. | 27% |  |  |  |  | Yes (1-23)  Yes (1-23) |
| PP4080 | TssK2 | 445 a.a. | PA1667  (HsiJ2) 35% | 91% | COG3522/pfam05936 (DUF876)/ TIGR03353 (VI_chp_4) | 1. Cytoplasmic  2. Periplasm | 0 | No  No |
| PP2617 | TssK3 | 445 a.a. | 35% |  |  |  |  |  |
| PP4081 | TssL2 | 310 a.a. | PP1668(DotU2)34% | 85% | COG3455/ pfam09850 (DUF2077)/ TIGR03349 (IV_VI_DotU) | 1. Unknown  2. Inner Membrane | 1 | No  No |
| PP2616 | TssL3 | 289 a.a. | 34% |  |  |  |  |  |
| PP4082 | TssD2/Hcp2 | 171 a.a. | PA1512  (Hcp2)  58% | 99% | COG3157 (Hcp)/ pfam05638 (DUF796)/ TIGR03344 (VI_effect_Hcp1) | 1. Extracellular  2. Extracellular | 0 | No  No |
| PP2615 | TssD3/Hcp3 | 171 a.a. | 58% |  |  |  |  |  |
| PP4083 | TssI2/VgrG2 (partial) | 361/659 a.a. | PA1511 (VgrG2a)  49% | 76% | COG3501 (VgrG)/ pfam05954 (Phage_GPD)/ TIGR03361 (VI_Rhs_Vgr) | 1. Cytoplasmic  2. Cytoplasmic | 0 | No  No |
| PP2614 | TssI3/VgrG3 | 722 a.a. | 53% |  |  |  |  |  |
|  |  |  |  |  |  |  |  |  |
|  |  |  |  |  |  |  |  |  |
| PP2613 | Fha3 | 316 a.a. |  |  | pfam13503 (DUF4123) | 1. Unknown  2. Cytoplasmic | 0 | No  No |
| PP2612 | Tke5 | 996 a.a. |  |  | -- | 1. Inner Membrane  2. Unknown | 5 | No  Yes (1-35) |
| PP2611 | Tki5 | 319 a.a. |  |  | pfam11746 (DUF3303) | 1. Inner Membrane  2. Inner Membrane | 4 | No  Yes (1-21) |
| PP2610 | Tsp5 | 85 a.a. |  |  | COG4101/pfam05488 (PAAR_motif) | 1. Unknown  2. Cytoplasmic | 0 | No  No |
|  |  |  |  |  |  |  |  |  |
|  |  |  |  |  |  |  |  |  |
| PP4084 | EagR2 | 143 a.a. |  |  | COG5435/ pfam08786 (DUF1795) | 1. Unknown  2. Cytoplasmic | 0 | No  No |
| PP4085 | Tke4 | 1530 a.a. |  |  | Pfam05488 (PAAR_motif) + COG3209(RhsA)/pfam03527(RHS)/TIGR03696 (Rhs_assc_core) + pfam15652 (Tox-SHH) | 1. Unknown  2. Inner Membrane | 3 | No  Yes (1-28) |
| **PP4085.1** | Tki4 | 161 a.a. |  | PP4094  96% | SM000860(SMI1/KNR4 family) Imm-SUKH | 1. Unknown  2. Extracellular | 0 | No  No |
| **PP4085.2** |  | 143 a.a. |  | PP4094.1  73% | -- | 1. Cytoplasmic  2. Cytoplasmic | 0 | No  No |
| PP4086 |  | 187 a.a. |  |  | -- | 1. Inner Membrane  2. Cytoplasmic | 1 | No  No |
| PP4087 |  | 50 a.a. |  |  | -- | 1.Unknown  2. Unknown | 0 | No  No |
| **PP4087.1** | Partial | 100 a.a. |  |  | TIGR03696 (Rhs_assc_core) | 1. Unknown  2. Cytoplasmic | 0 | No  No |
| **PP4087.2** | Partial | 140 a.a. |  |  | pfam15428 (Imm14) | 1. Unknown  2. Cytoplasmic | 0 | No  No |
| PP4088 | Partial | 491a.a. |  | PP4085  95% | pfam05593 (RHS_repeat) | 1. Unknown  2. Extracellular | 0 | No  No |
| PP4089 | Partial | 422 a.a. |  | PP4085  82% | pfam03257 (RHS) | 1. Unknown  2. Extracellular | 0 | No  No |
| **PP4089.1** |  | 153 a.a. |  |  | -- | 1.Cytoplasmic  2. Cytoplasmic | 0 | No  No |
| PP4090 | Partial | 302 a.a. |  | PP4085  98% | pfam05593 (RHS_repeat) | 1. Unknown  2. Extracellular | 0 | No  No |
| PP4091 | ISPpu15 Orf2 |  |  |  | COG3436/ pfam03050 (DDE_Tnp_IS66) |  |  |  |
| PP4092 | ISPpu15 Orf1 |  |  |  | COG3436/ pfam05717 (TnpB_IS66) |  |  |  |
| PP4093 | Partial | 198 a.a. |  | PP4085  57% | COG3209 (RhsA)/TIGR03696 (tRNA_nuclease_WapA) | 1. Unknown  2. Cytoplasmic | 0 | No  No |
| **PP4093.1** | Partial | 212 a.a. |  |  | P: Hypothetical protein YwqG (pdb: d1pv5a) (143-212 a.a.) C: 93% | 1. Unknown  2. Outer Membrane | 0 | No  No |
| PP4094 | Tki4b | 161 a.a. |  | PP4085.1 96% | SM000860(SMI1/KNR4 family) Imm-SUKH | 1. Cytoplasmic  2. Extracellular | 0 | No  No |
| **PP4094.1** |  | 101 a.a. |  | PP4085.2  73% | Pfam09827 (CRISPR_Cas2) | 1. Unknown  2. Cytoplasmic | 0 | No  No |
| PP4095 | Partial | 91 a.a. |  |  | pfam05954 (Phage GPD) | 1. Unknown  2. Cytoplasmic | 0 | No  No |

a.a.: amino acids.

Newly annotated proteins are in bold

Partial proteins or those with premature stop codon are underline

a: Structural-based homology prediction using the Protein Homology/analogy Recognition Engine (Phyre) server (Kelley, et al., 2009). C stands for Confidence.

b: The cellular localization is based on prediction by PSORTb

(http://www.psort.org/psortb/index.html).

c: The cellular localization is based on prediction by SOSUI GramN

(http://bp.nuap.nagoya-u.ac.jp/sosui/sosuigramn/sosuigramn_submit.html).

d: The prediction of transmembrane domains was determined by TMHMN (http://www.cbs.dtu.dk/services/TMHMM/)

e: The prediction of signal peptides was by SignalP

(http://www.cbs.dtu.dk/services/SignalP/).

f: The prediction of signal peptides was by SOSUIsignal (http://bp.nuap.nagoya-u.ac.jp/sosui/sosuisignal/sosuisignal_submit.html).
