# Supplementary material for: The Pseudomonas putida T6SS is a plant warden against phytopathogens
Source: ISME J. 2017 Jan 3;11(4):972–87. doi: 10.1038/ismej.2016.169 (PMC5363822; doi:10.1038/ismej.2016.169)
Supplement: Supplementary Table S6 [file ismej2016169x13.docx]

**Table S6:** Characteristics of proteins encoded by orphan *hcp* and *vgrG* gene clusters

| Locus name | Gene name  Protein name | Identities | Conserved Domains  COG/pfam/TIGR  (Short Name)/Phyre^a^ | Molecular weight  (KDa)/pI^b^ | Predicted cellular location^c, d^ | Transmembrane helices^e^ | Predicted signal peptide^f, g^ | Predicted non-classically secreted protein^h^ |
| --- | --- | --- | --- | --- | --- | --- | --- | --- |
|  |  |  |  |  |  |  |  |  |
| **Orphan Hcp4 cluster** | | | | | | | | |
| PP0646 | Tke6 |  | COG4101/pfam05488 (PAAR_motif) + P: Colicin n (242-460 a.a.)  C: 87.6% | 466 a.a.  48.7/5.8 | 1. Unknown  2. Inner membrane | 2 | No  No | No |
| PP0647 | Tki6 |  | -- | 129 a.a.  14.6/9.46 | 1. Inner membrane  2. Inner membrane | 3 | No  Yes  (1-37) | No |
| PP0655 | Hcp4 (Partial) | PA0085 (Hcp1)  24/80 (30%) | COG3157/pfam05638 (DUF796) | 108 a.a.  11.7/4.2 | 1. Unknown  2. Extracellular | 0 | No  No | Yes |
|  |  |  |  |  |  |  |  |  |
| **Orphan Hcp5 cluster** | | | | | | | | |
| **PP4884.1** | Tki7 | PP5238.2 43/ 104 (41%) | -- | 124 a.a.  14.3/10.3 | 1. Inner membrane  2. Inner membrane | 2 | No  No | No |
| PP4885 | Tke7 | PP5238.1 152/250 (61%) | P: Colicin s4 (57-314 a.a.) C: 95% | 323 a.a.  34/6.4 | 1. Cytoplasmic  2. Unknown | 0 | No  No | No |
| PP4886 | Hcp5 | PA0085 (Hcp1)  47/157 (30%)  PP5238 (Hcp6) 150/162 (93%) | COG3157/pfam05638 (DUF796)/TIGR03344 (VI_effect_Hcp1) | 181 a.a.  19.8/8.4 | 1. Extracellular  2. Unknown | 0 | No  No | Yes |
|  |  |  |  |  |  |  |  |  |
| **Orphan Hcp6 cluster** | | | | | | | | |
| PP5238 | Hcp6 | PA0085 (Hcp1)  47/157 (30%)  PP4886 (Hcp5) 150/162 (93%) | COG3157/pfam05638 (DUF796)/TIGR03344 (VI_effect_Hcp1) | 162 a.a.  17.5/7.2 | 1. Extracellular  2. Cytoplasmic | 0 | No  No | Yes |
| **PP5238.1** | Tke8 | PP4885 (Tke7) 152/250 (61%) | P: Colicin n (43-258 a.a.)  C: 68% | 260 a.a.  26.9/7.8 | 1. Cytoplasmic  2. Inner membrane | 0 | No  No | No |
| **PP5238.2** | Tki8 | PP4884.1 (Tki7)  43/104 (41%) | -- | 149 a.a.  17/9.3 | 1. Inner membrane  2. Inner membrane | 3 | No  No | No |
|  |  |  |  |  |  |  |  |  |
| **Orphan VgrG4 cluster** | | | | | | | | |
| PP3385 | TssL4 | PA1668 (DotU2)  69/207 (33%) | COG3455/pfam09850 (DUF2077)/TIGR03349 (IV_VI_DotU) | 282 a.a.  32.4/6 | 1. Cytoplasmic  2. Inner membrane | 1 | No  No | No |
| PP3386 | VgrG4 | PA1511 (VgrG2a)  270/506 (53%) | COG3501 (VgrG)/ pfam05954 (Phage_GPD)/ TIGR03361 (VI_Rhs_Vgr) | 725 a.a.  82/6.1 | 1. Cytoplasmic  2. Cytoplasmic | 0 | No  No | No |
| PP3387 | Tap4 | PA1854  38/134 (28%) | pfam13503 (DUF4123) | 315 a.a.  34.9/7.7 | 1. Unknown  2. Cytoplasmic | 0 | No  No | No |
| PP3388 | Tke9 |  | -- | 890 a.a.  99.2/6.8 | 1. Unknown  2. Inner membrane | 2 | No  No | No |
| PP3389 | Tki9 |  | -- | 385 a.a.  43.3/10.4 | 1. Inner membrane  2. Inner membrane | 3 | No  No | No |
|  |  |  |  |  |  |  |  |  |
| **Orphan VgrG5 cluster** | | | | | | | | |
| PP4045 | Tsp10 | PA0093 (Tke6)  18/46 (39%) | COG4104/pfam05488 (PAAR_motif) | 88 a.a.  8.9/6 | 1. Unknown  2. Cytoplasmic | 0 | No  Yes  (1-13) | Yes |
| PP4046 | Tki10b | PA2201  35/143 (24%)  PP4047 (Tki10a)  120/320 (38%) | pfam08928 (DUF1910) Imm PA2201 | 320 a.a.  37.1/6.5 | 1. Cytoplasmic  2. Cytoplasmic | 0 | No  No | No |
| PP4047 | Tki10a | PA2201 44/152 (29%)  PP4046 (Tki10b)  120/320 (38%) | pfam08929 (DUF1911) Imm PA2201 | 318 a.a.  37.1/5.3 | 1. Cytoplasmic  2. Cytoplasmic | 0 | No  No | No |
| PP4048 | Tke10 |  | Tox-REase-1 (Zhang et al. 2012) | 507 a.a.  54.8/8.5 | 1. Unknown  2. Inner Membrane | 1 | No  No | No |
| PP4049 | VgrG5 | PA1511 (VgrG2a)  259/509 (51%) | COG3501 (VgrG)/ pfam05954 (Phage_GPD)/ TIGR03361 (VI_Rhs_Vgr) | 771 a.a.  85.6/6 | 1. Cytoplasmic  2. Cytoplasmic | 0 | No  No | No |

a.a.: amino acids

Newly annotated proteins are in bold

Partial proteins or those with premature stop codon are underline

a: Structural-based homology prediction using the Protein Homology/analogy Recognition Engine (Phyre) server (Kelley, et al., 2009). C stands for Confidence.

b: The molecular weight and isoelectric point (pI) are based on prediction by the software ExPASy (http://www.expasy.ch/tools/pi_tool.html).

c: The cellular localization is based on prediction by PSORTb

(http://www.psort.org/psortb/index.html).

d: The cellular localization is based on prediction by SOSUIGramN

(http://bp.nuap.nagoya-u.ac.jp/sosui/sosuigramn/sosuigramn_submit.html).

e: The prediction of transmembrane domains was determined by TMHMN (http://www.cbs.dtu.dk/services/TMHMM/)

f: The prediction of signal peptides was by use of SignalP

(http://www.cbs.dtu.dk/services/SignalP/).

g: The prediction of signal peptides was determined by SOSUIsignal (http://bp.nuap.nagoya-u.ac.jp/sosui/sosuisignal/sosuisignal_submit.html).

h: The prediction was determined by SecretomeP (http://www.cbs.dtu.dk/services/SecretomeP/).
